# Separable functions of Tof1/Timeless in intra-S-checkpoint signalling, replisome stability and DNA topological stress

**DOI:** 10.1093/nar/gkaa963

**Published:** 2020-11-09

**Authors:** Rose Westhorpe, Andrea Keszthelyi, Nicola E Minchell, David Jones, Jonathan Baxter

**Affiliations:** Genome Damage and Stability Centre, School of Life Sciences, Science Park Road, University of Sussex, Falmer, Brighton, East Sussex BN1 9RQ, UK; Genome Damage and Stability Centre, School of Life Sciences, Science Park Road, University of Sussex, Falmer, Brighton, East Sussex BN1 9RQ, UK; Genome Damage and Stability Centre, School of Life Sciences, Science Park Road, University of Sussex, Falmer, Brighton, East Sussex BN1 9RQ, UK; Genome Damage and Stability Centre, School of Life Sciences, Science Park Road, University of Sussex, Falmer, Brighton, East Sussex BN1 9RQ, UK; Genome Damage and Stability Centre, School of Life Sciences, Science Park Road, University of Sussex, Falmer, Brighton, East Sussex BN1 9RQ, UK

## Abstract

The highly conserved Tof1/Timeless proteins minimise replication stress and promote normal DNA replication. They are required to mediate the DNA replication checkpoint (DRC), the stable pausing of forks at protein fork blocks, the coupling of DNA helicase and polymerase functions during replication stress (RS) and the preferential resolution of DNA topological stress ahead of the fork. Here we demonstrate that the roles of the *Saccharomyces cerevisiae* Timeless protein Tof1 in DRC signalling and resolution of DNA topological stress require distinct N and C terminal regions of the protein, whereas the other functions of Tof1 are closely linked to the stable interaction between Tof1 and its constitutive binding partner Csm3/Tipin. By separating the role of Tof1 in DRC from fork stabilisation and coupling, we show that Tof1 has distinct activities in checkpoint activation and replisome stability to ensure the viable completion of DNA replication following replication stress.

## INTRODUCTION

The faithful replication of the genome by the DNA replication machinery is hindered by a range of exogenous and endogenous factors that are capable of disrupting replication forks. Such cellular events are a prominent feature of the early stages of cancer and have been collectively referred to as replication stress (RS) (1). RS occurs when either the helicase or polymerase activities of the replisome are impeded. Potential impediments include chemical changes to the DNA, stable DNA-binding protein complexes, nucleotide deficiency and DNA topological stress (2,3).

On encountering RS, the replisome is thought to be stabilised at the replication fork until the impeding context is removed or bypassed and replication restarted (4). A failure to stabilise the replisome is associated with erroneous processing of the replication fork, leading to either toxic recombination intermediates or disruption of replication restart.

The activation of checkpoint pathways is essential for replication fork stabilisation and restart (5). Following RS, ATR type kinases (Mec1 in *Saccharomyces cerevisiae*) are activated by the accumulation of excessive RPA-coated single stranded DNA (6,7). ATR/Mec1 then acts with mediator proteins to activate effector kinases which promote fork stability, regulate nucleotide metabolism, and inhibit the firing of further replication origins (8). The mediator proteins required for effector kinase activation can act either at the replication fork, commonly referred to as the DNA replication checkpoint (DRC), or independently of the fork, referred to as the DNA damage checkpoint (DDC). Mediator proteins in the DRC include the replisome proteins Mrc1/Claspin (*Sc* Mrc1) (9) and Tof1/Swi1/Timeless (*Sc* Tof1) (10).

Tof1 appears to act as a nexus for various processes relating to both stabilising the replication fork during RS and ensuring faithful chromosome inheritance. In addition to its role in activating effector kinases, Tof1, Mrc1 and the Tof1 interacting protein Csm3/Swi3/Tipin (*Sc* Csm3) are required to stimulate DNA replication *in vivo* and *in vitro* (11–14), and to couple helicase and polymerase activities (15–19). Independently of Mrc1, the Tof1-Csm3 complex is also required for pausing of replication forks at stable protein-DNA barriers (11,20,21) and to focus the action of topoisomerases ahead of the fork to prevent excessive fork rotation during DNA replication (22). This latter activity has been linked to the observation that the C terminus of Tof1 and the type IB topoisomerase Top1 interact (23), potentially ensuring a relative enrichment of Top1 at the replication fork (3,22). Consistent with this model, Tof1 is required to recruit Top1 to replicating regions *in vivo* (24). In addition, both *tof1Δ* and *csm3Δ* (but not *mrc1Δ*) cells are acutely sensitive to the chemotherapeutic drug camptothecin (CPT), which stabilises the covalently DNA-bound intermediate of Top1 (25–27). The potential linkage between the Tof1-Csm3-dependent activities of fork pausing, resolving DNA topological stress and resistance to CPT treatment is unclear.

To attempt to define the molecular relationships between the processes that involve Tof1, we have generated a series of truncations across the C terminus of Tof1 and assessed whether the truncated proteins are capable of maintaining the functions of Tof1 in activating the DRC, replication fork pausing, helicase-polymerase coupling and preventing excessive fork rotation in response to DNA topological stress. We find that the far C terminus is required to resolve DNA topological stress but not for fork pausing, replisome coupling, CPT resistance or DRC activation signalling. The near C terminus is required for fork pausing, fork coupling and CPT resistance but not DRC signalling. This region is also required for the interaction between Tof1 and Csm3. We also find that the N terminal half of Tof1 alone is sufficient for DRC signalling. Our data indicate a modular functionality for Tof1 with the N terminal half supporting the DRC, the far C terminus recruiting Top1 to the fork, and the interaction between Tof1 and Csm3 closely linked to fork pausing, replisome coupling and CPT resistance.

## MATERIALS AND METHODS

### Yeast strains, plasmids and primers

All yeast strains in this study were generated in the W303 background (*ade2–1 ura3–1 his3–11, trp1–1 leu2–3, can1–100*) and are listed in Table 1.

**Table 1.** Yeast strains used in this study

No.	Name	Genotype	Source
1991	*wt*	*MATa, ade2–1 his3–11 leu2–3 trp1–1 ura3–1 can1–100, UBR1::GAL1–10-Ubiquitin-M-LacI fragment-Myc-UBR1 (HIS3), leu2–3::pCM244 (CMVp-tetR’-SSN6, LEU2) x3*	S. Tanaka, J. F. Diffley, *Nature Cell Biology*. **4**, 198–207 (2002).
1993	*tof1Δ*	*1991 + TOF1::hphMX*	This study
1528	*TOF1*	*1991 +* *TOF1::TOF1-codon-optimised-wildtype (NATNT2)*	This study
1549	*tof1 627*	*1991 + TOF1::tof1-codon-optimised-627–1238Δ (NATNT2)*	This study
1596	*tof1 762*	*1991 + TOF1::tof1-codon-optimised-762–1238Δ (NATNT2)*	This study
1589	*tof1 830*	*1991 + TOF1::tof1-codon-optimised-830–1238Δ (NATNT2)*	This study
1546	*tof1 997*	*1991 + TOF1::tof1-codon-optimised-997–1238Δ (NATNT2)*	This study
1537	*tof1 1182*	*1991 + TOF1::tof1-codon-optimised-1182–1238Δ (NATNT2)*	This study
2188	*TOF1 wt TAP*	*1991 + endogenous TOF1 tagged with TAP (KanMX)*	This study
2182	*TOF1* *TAP*	*1991 + TOF1::tof1-codon-optimised-wildtype-TAP (KanMX)*	This study
2183	*tof1 627 TAP*	*1991 + TOF1::tof1-codon-optimised-627–1238Δ-TAP (KanMX)*	This study
2184	*tof1 762 TAP*	*1991 + TOF1::tof1-codon-optimised-762–1238Δ-TAP (KanMX)*	This study
2185	*tof1 830 TAP*	*1991 + TOF1::tof1-codon-optimised-830–1238Δ-TAP (KanMX)*	This study
2186	*tof1 997 TAP*	*1991 + TOF1::tof1-codon-optimised-997–1238Δ-TAP (KanMX)*	This study
2187	*tof1 1182 TAP*	*1991 + TOF1::tof1-codon-optimised-1182–1238Δ-TAP (KanMX)*	This study
2288	*TOF1 wt mrc1–3FLAG*	*1528 + Mrc1–3FLAG (NATNT2)*	This study
2191	*TOF1 wt-TAP mrc1–3FLAG*	*2182 + Mrc1–3FLAG (NATNT2)*	This study
2192	*tof1 627-TAP mrc1–3FLAG*	*2183 + Mrc1–3FLAG (NATNT2)*	This study
2194	*tof1 762-TAP mrc1–3FLAG*	*2184 + Mrc1–3FLAG (NATNT2)*	This study
2195	*tof1 830-TAP mrc1–3FLAG*	*2185 + Mrc1–3FLAG (NATNT2)*	This study
2196	*tof1 997-TAP mrc1–3FLAG*	*2186 + Mrc1–3FLAG (NATNT2)*	This study
2197	*tof1 1182-TAP mrc1–3FLAG*	*2187 + Mrc1–3FLAG (NATNT2)*	This study
2465	*GAL-HA-TOF1 wt*	*1991 + TOF1:: GAL-S-HA-TOF1-codon-optimised-wt (TRP)*	This study
2466	*GAL-HA-tof1 627*	*1991 + TOF1:: GAL-S-HA-TOF1-codon-optimised-627–1238Δ (TRP)*	This study
2467	*GAL-HA-tof1 997*	*1991 + TOF1:: GAL-S-HA-TOF1-codon-optimised-997–1238Δ (TRP)*	This study
2468	*GAL-HA-tof1 1182*	*1991 + TOF1:: GAL-S-HA-TOF1-codon-optimised-1182–1238Δ (TRP)*	This study
2414	*wt TOF1* *(*diploid*)*	*MAT a/alpha (diploid)* *homozygous: ade2–1 his3–11 leu2–3 trp1–1 ura3–1 can1–100, UBR1::GAL1–10-Ubiquitin-M-LacI fragment-Myc-UBR1 (HIS3), leu2–3::pCM244 (CMVp-tetR’-SSN6, LEU2) x3*,*heterozygousTOF1::tof1-codon-optimised-wildtype (NATNT2)*	This study
2420	*tof1 627 TAP top1-MYC (*diploid*)*	*MAT a/alpha (diploid) heterozygous: ade2–1 his3–11 leu2–3 trp1–1 ura3–1 can1–100, UBR1::GAL1–10-Ubiquitin-M-LacI fragment-Myc-UBR1 (HIS3), leu2–3::pCM244 (CMVp-tetR’-SSN6, LEU2) x3 TOF1::tof1-codon-optimised-627–1238Δ-TAP (KanMX) Top1–9MYC* *(hphMX)*	This study
2463	*tof1 997 TAP top1-myc (*diploid*)*	*MAT a/alpha (diploid)* *homozygous: ade2–1 his3–11 leu2–3 trp1–1 ura3–1 can1–100, UBR1::GAL1–10-Ubiquitin-M-LacI fragment-Myc-UBR1 (HIS3), leu2–3::pCM244 (CMVp-tetR’-SSN6, LEU2)* *x3, heterozygous TOF1::tof1-codon-optimised-997–1238Δ-TAP (KanMX) Top1–9MYC* *(hphMX)*	This study
2416	*tof1 1182 TAP top1-myc (*diploid*)*	*MAT a/alpha (diploid)* *homozygous: ade2–1 his3–11 leu2–3 trp1–1 ura3–1 can1–100, UBR1::GAL1–10-Ubiquitin-M-LacI fragment-Myc-UBR1 (HIS3), leu2–3::pCM244 (CMVp-tetR’-SSN6, LEU2)* *x3, heterozygousTOF1::tof1-codon-optimised-1182–1238Δ-TAP (KanMX) Top1–9MYC* *(hphMX)*	This study
1988	*csm3Δ*	*1991 + CSM3:: NATNT2*	This study
684	*tof1Δ rad9Δ*	*1993 + rad9::NATNT2*	This study
2405	*csm3Δ rad9Δ*	*1988 + rad9::KanMX*	This study
1718	*TOF1 wt rad9Δ*	*1528 + rad9::KanMX*	This study
1719	*tof1 627 rad9Δ*	*1549 + rad9::KanMX*	This study
1777	*tof1 762 rad9Δ*	*1596 + rad9::KanMX*	This study
1767	*tof1 830 rad9Δ*	*1589 + rad9::KanMX*	This study
1722	*tof1 997 rad9Δ*	*1546 + rad9::KanMX*	This study
1769	*tof1 1182 rad9Δ*	*1537 + rad9::KanMX*	This study
*1762*	*TOF1 wt top2–4 pRS316*	*1528 + top2–4*,*pRS316* (URA)	This study
*1610*	*tof1 627 top2–4 pRS316*	*1549 + top2–4*,*pRS316* (URA)	This study
2290	*tof1 762 top2–4 pRS316*	*1596 + top2–4*,*pRS316* (URA)	This study
1717	*tof1 830 top2–4 pRS316*	*1589 + top2–4*,*pRS316* (URA)	This study
1763	*tof1 997 top2–4 pRS316*	*1546 + top2–4*,*pRS316* (URA)	This study
1611	*tof1 1182 top2–4 pRS316*	*1537 + top2–4*,*pRS316* (URA)	This study
*1731*	*wt pRS426-RFB*	*1991 + pRS426–1xRFB (URA)*	This study
*1730*	*tof1Δ* pRS426-RFB	*1993 + pRS426–1xRFB (URA)*	This study
*1732*	*TOF1 wt pRS426-RFB*	*1528 + pRS426–1xRFB (URA)*	This study
*1739*	*tof1 627 pRS426-RFB*	*1549 + pRS426–1xRFB (URA)*	This study
*2333*	*tof1 762 pRS426-RFB*	*1596 + pRS426–1xRFB (URA)*	This study
*1740*	*tof1 830 pRS426-RFB*	*1589 + pRS426–1xRFB (URA)*	This study
*1733*	*tof1 997 pRS426-RFB*	*1546 + pRS426–1xRFB (URA)*	This study
*1741*	*tof1 1182 pRS426-RFB*	*1537 + pRS426–1xRFB (URA)*	This study

See Table 2 for all plasmids used in this study.

**Table 2.** Plasmids used in this study

Plasmid name	Source	Used for
pRS306/Tof1- Gal-CBP-Csm3	Yeeles *et al.*, 2017	Generation of pBAK004 containing *TOF1*-codon optimised sequence upstream of NATNT2 selection marker
PBAK004 (TOF1-NATNT2)	This study	Used as template for site-directed mutagenesis of *TOF1* codon-optimised gene
tof1 627-NATNT2	This study	Used as PCR template to generate fragment: *tof1*-codon-optimised-627–1238Δ-NATNT2 for yeast transformation into endogenous *TOF1* locus
tof1 762-NATNT2	This study	Used as PCR template to generate fragment: *tof1*-codon-optimised-762–1238Δ-NATNT2 for yeast transformation into endogenous *TOF1* locus
tof1 830-NATNT2	This study	Used as PCR template to generate fragment: *tof1*-codon-optimised-830–1238Δ-NATNT2 for yeast transformation into endogenous *TOF1* locus
tof1 997-NATNT2	This study	Used as PCR template to generate fragment: *tof1*-codon-optimised-997–1238Δ-NATNT2 for yeast transformation into endogenous *TOF1* locus
tof1 1182-NATNT2	This study	Used as PCR template to generate fragment: *tof1*-codon-optimised-1182–1238Δ-NATNT2 for yeast transformation into endogenous *TOF1* locus
pRS316	Sikorski, R. S. and Hieter, P. (1989)	Transformed into *tof1 top2–4* strains for fork rotation/catenation assay
4XRFB	Luis Aragon Lab (unpublished)	Used as PCR template to amplify 1xRFB sequence for cloning into pRS426
pRS426-RFB	This study	Transformed into *tof1 top2–4* strains for fork pausing assay

Generation of yeast strains expressing truncated forms of Tof1 was carried out in two steps. First, codon optimised *TOF1* gene was cut out from a pRS306/Tof1-Gal-CBP-Csm3 plasmid (gift from Diffley lab) (12) by NotI digestion and cloned into pFA6-natNT2. The codon optimised TOF1 was then mutagenised using QuikChange Lightning Site Directed Mutagenesis kits (Agilent, 210518) according to manufacturer's instructions, with primer sets designed to incorporate premature stop codons into the *TOF1* open reading frame. Mutagenesis was confirmed by sequencing before PCR amplification and insertion of the mutagenised sequence and Nourseothricin resistance gene into the endogenous *TOF1* locus of yeast cells using lithium acetate transformation.

Plasmid pRS426-RFB was generated by a two-fragment Gibson assembly. Specifically, a single RFB site was PCR-amplified from plasmid 4xRFB (gift from Luis Aragon) corresponding to the sequence from *Saccharomyces cerevisiae* S288C Chromosome XII: 459799–460920. This was assembled with *PfoI*-linearized pRS426 using the NEBuilder^®^ HiFi DNA Assembly Cloning Kit (New England Biolabs, E5520S).

### Media and cell-cycle synchronisation

For plasmid catenation experiments in *top2–4* pRS316-containing strains, cells were grown to mid-log phase in synthetic complete media without uracil +2% glucose, before being re-suspended in YP 2% Glucose (YPD). Cells were arrested in G1 by addition of 10 μg/ml alpha factor peptide (Genscript) for 1.5 h, after which a second dose of alpha factor (5 μg/ml) was added. When >90% of cells were unbudded, cultures were shifted to the restrictive temperature for *top2–4* (37°C) 1 h before release into S-phase achieved by washing three times with YPD at 37°C. Time 0 indicates the time from addition of the first wash. 50 μg/ml nocodazole was added to cultures at 45 min to prevent mitotic entry, and at 80 min from release 10 ml samples for 2D gel and Southern blotting analysis were collected by centrifugation and snap-freezing the pellets in liquid nitrogen.

For fork pausing experiments cells containing pRS426-RFB were grown to mid-log phase in synthetic complete media without uracil +2% glucose. 10 ml cultures were collected by centrifugation and snap-freezing the pellets in liquid nitrogen.

For experiments involving treatment with HU, cells were grown in YPD to mid-log phase before either HU being added to 200 mM for 2 h (for Rad53 activation experiments in Figure 5A) or for experiments started with a G1 arrest, mid-log cells were treated with 10 μg/ml alpha factor peptide. To release cultures into the cell cycle, when cells were >90% unbudded, cultures were washed 3 times in YPD and re-suspended after the third wash in YPD containing 200 mM HU. Time 0 was designated as the time from the first wash. Time points were taken as indicated. For release from HU experiments, HU-containing media was washed off of cells by washing three times with YPD before re-suspending in YPD. Time 0 is taken as the time from the first YPD wash.

For RPA ChIP experiments cells were grown in YP +2% raffinose to mid-log phase at 25°C, before being arrested with 10 μg/ml alpha factor peptide. After 1 h 45 min 2% galactose and an additional 5 μg/ml alpha factor was added. After 2 h, when cells were >90% unbudded, 25 μg/ml doxycycline was added. Fifteen minutes after doxycycline addition the temperature was switched to 37°C and incubated for 1 h. Cells were then released by washing 3 times with pre-heated YP 2% raffinose 2% galactose with 25 μg/ml doxycycline and resuspended in the same media supplemented with 200 mM HU. Time 0 was taken as the time from the first wash. Samples were then incubated for 1 h at 37°C before being fixed by resuspending in YP + 1% formaldehyde (Sigma) for 45 min at 25°C. 125 mM glycine was then added followed by a 5 min incubation at 25°C. Cells were washed with PBS before being pelleted and snap-frozen in liquid nitrogen.

### TCA extraction

10 ml of mid-log yeast cultures were centrifuged at 3500 rpm for 5 min and the resulting cell pellets were snap-frozen in liquid nitrogen before storing at −80°C. All further steps were carried out on ice, and centrifugation and homogenisation steps at 4°C. 200 μl of 20% TCA was added to thawed cell pellets and the cell suspension was transferred to screw-cap tubes containing 500 μl of 0.5 mm zirconia/silica beads (BioSpec Products). Cells were homogenised using a FastPrep-24 (MP Biomedicals) on max speed (6.5 m/s) for 4 × 1 min pulses, with 1 min on ice in between pulses. Beads were separated from the lysate by piercing the tubes and centrifugation of the mixtures into fresh tubes at 3000 rpm for 2 min. Beads were washed once with 600 μl 5% TCA and centrifuged again into tubes containing the cell lysate. Cell extract/TCA mixtures were then centrifuged at 13 000 rpm for 5 min before removing all TCA from the resulting pellets. This step was repeated once to ensure complete removal of all TCA. To the pellets 200 μl of 1× sample buffer was added before boiling samples for 5 min. Samples were spun at 13 000 rpm for 5 min and the resulting supernatants were collected and stored at −20°C for SDS-PAGE and western blotting analysis.

### SDS-PAGE and western blotting

Protein extracts prepared by TCA extraction were run on either 8%, 10% or 12% SDS-PAGE gels before being wet-transferred to nitrocellulose membranes at 50V for 90 min, 4°C. Proteins were visualised by staining in Ponceau for 30 seconds before membranes were blocked in 5% milk (Marvel) PBS 0.2% Tween-20 (PBS-T). All primary antibodies were diluted in 5% milk PBS-T and incubated overnight at 4°C. In between primary and secondary antibody incubations membranes were washed 3 times with PBS-T for 15 min. Secondary antibodies were diluted in 5% milk PBS-T and incubated with membranes for 1 h at room temperature. Proteins were detected using Western Lightning Plus-ECL (Perkin-Elmer, NEL104001EA) and images were acquired on an ImageQuant LAS4000 system (GE Healthcare). Densitometry analysis was carried out using ImageQuant TL software.

Primary antibodies used for immunoblotting in this study: OctA-Probe Antibody 1:1000 (for detecting FLAG-tagged proteins) (sc-166355), anti-Csm3 (28), anti-PAP 1:1000 (for detecting TAP-tagged proteins) (Sigma-Aldrich, P1291), anti-Rad53 1:2000 (abcam ab104232), anti-HA 1:1000 (Roche 12CA5), anti-c-Myc 1:1000 (sc-9E10). Secondary antibodies used in this study: anti-mouse-HRP 1:1000 (Dako P0260), anti-rabbit-HRP 1:1000 (Dako P0448) and anti-sheep-HRP 1:10 000 (Sigma-Aldrich A3415).

### TAP pulldowns

200 ml of mid-log yeast cultures were centrifuged for 5 min at 3500 rpm and washed once in ice-cold PBS. The resulting cell pellet was snap-frozen in liquid nitrogen and stored at −80°C before proceeding. Frozen pellets were re-suspended in 0.5 ml of ice-cold lysis buffer (50 mM HEPES pH 7.5, 300 mM KCl, 5 mM EDTA, 10 mM MgOAc, 10% glycerol, 80 mM Beta-glycerophosphate, 0.01% Triton X-100, 1 protease inhibitor tablet (cOmplete™, EDTA-free Protease Inhibitor Cocktail, Roche) and 1 phosphatase inhibitor tablet (PhosSTOP™, Roche) per 50ml of lysis buffer) in screw-cap tubes. 500 μl of zirconia/silica beads (BioSpec Products) were added and the cell suspensions were homogenised using the FastPrep-24 (MP Biomedicals) on max speed (6.5 m/s) for 4 pulses of 1 min each, with 3 min on ice in between each pulse. Beads were separated from the lysate by piercing the tubes and centrifugation into fresh tubes at 3000 rpm for 2 min. For Csm3 pulldowns in Figure 7 the supernatants were immediately cleared by centrifugation at 13 000 rpm for 30 min at 4°C before addition of beads. For Top1 pulldowns in Supplementary Figure S2 the lysate was first treated with 100 U benzonase (Merck) for 40 min at 4°C before being cleared by centrifugation at 13 000 rpm for 30 min at 4°C. Cleared supernatants were transferred to fresh tubes before starting the pulldown. 20 μl of IgG Sepharose 6 Fast Flow affinity resin (GE-Healthcare, #17096901) for each pulldown were pre-washed 3 times in cold lysis buffer before being added to the cleared cell lysates. The lysate/bead mixtures were incubated at 4°C on a rotating platform for 2 h and subsequently washed 4 times with 1 ml ice cold lysis buffer. For Csm3 pulldowns in Figure 7 beads were washed in Poly-Prep^®^ Chromatography Columns (Bio-Rad #7311550) and the bound fraction was eluted in 0.5 ml of 0.2 M glycine pH 3.0. For Top1 pulldowns in Supplementary Figure S2 the beads were washed in microcentrifuge tubes 4 times for 5 min on a rotating platform at 4°C, with centrifugation at 2000 rpm for 2 min between each wash to collect beads. The washed beads were then boiled for 5 min in 1× sample buffer to elute proteins. Input proteins were precipitated by TCA extraction before running samples on SDS-PAGE gels, with western blotting performed as described.

### DNA purification for Southern blotting

Frozen pellets were re-suspended in 0.4 ml of DNA Extraction Buffer (50 mM Tris–HCl pH 8.0, 100 mM NaCl, 10 mM EDTA, 1% SDS) along with 40 Units of lyticase (Sigma-Aldrich, L2524) and 5 μl 2-mercaptoethanol (Sigma Aldrich, 63689). Samples were incubated at 37°C for 5 min before addition of 450 μl phenol/chloroform/isoamylalcohol (25:25:1, Sigma-Aldrich) and mixing by rotation. Phase lock tubes (5 Prime, 2302800) were used to collect the aqueous layer, by centrifugation for 5 min at 12 000 rpm. DNA was ethanol precipitated by addition of roughly 2× volume of 100% EtOH and washed once in 70% EtOH before air-drying and solubilisation in 10 mM Tris pH 8.0.

For plasmid DNA catenation analysis of pRS316, purified DNA was nicked with Nb.Bsm1 (New England Biolabs, R0706S) according to manufacturer's instructions.

For fork pausing analysis of pRS426-RFB, purified DNA was digested with BamHI-HF (New England Biolabs, R3136S) and SnaBI (New England Biolabs, R0130L) or SnaBI alone according to manufacturer's instructions.

After nicking/digestion the DNA was precipitated, washed and solubilised as above with the addition of 300 mM Sodium Acetate pH 5.2 at the first ethanol addition.

### 2D gel electrophoresis of replication intermediates or catenated plasmid replication products

Purified DNA was separated in the first dimension by electrophoresis in 0.4% MegaSieve/MegaBase Agarose (Scientific Laboratory Supplies, H15608), 1× TBE (90 mM Tris, 90 mM boric acid, 10 mM EDTA).

For DNA catenation analysis of plasmid pRS316 from *top2–4* cells, first dimension gels were run at room temperature for 16–18 h at 30 V. A lane of the gel containing a small amount of each DNA sample was excised and stained in 0.5 μg/ml Ethidium Bromide 1× TBE to reveal extent of genomic DNA mobility. The remaining non-stained gel slices containing the plasmid were excised and embedded in 1.2% MegaSieve/MegaBase Agarose 1X TBE and run in the second dimension in 1× TBE at 4°C for 16–17 h, 120 V.

For analysis of paused replication intermediates from plasmid pRS426-RFB, first dimension gels were run at room temperature for 15.5 h at 30 V. First dimension gels were stained in 0.5 μg/ml Ethidium Bromide in 1× TBE and gel slices containing the replication intermediates were excised and embedded in 1% MegaSieve/MegaBase Agarose 1× TBE 0.3 μg/ml Ethidium bromide gels. Second dimension gels were run in 1× TBE at 4°C for 8 h, 120 V with re-circulation of the running buffer.

### Southern blotting

Following 2D electrophoresis, gels were washed sequentially in depurination buffer (0.125 M HCl), denaturation buffer (0.5 M NaOH, 1.5 M NaCl) and neutralisation buffer (0.5 M Tris–HCl, 1.5 M NaCl pH 7.5) with washes in ddH_2_O in-between each buffer. DNA was transferred onto Hybond-N+ membrane (GE Healthcare) by capillary action in 20× SCC (3 M NaCl, 350 mM NaOC trisodium citrate pH 7.0). Membranes were cross-linked using a UV Stratalinker 1800 (Stratagene) at 1200 J/m and subsequently blocked in hybridisation buffer (5× SSC, 5% Dextran sulphate (Sigma-Aldrich, D8906) 0.2% Tropix I-Block (Applied Biosystems, T2015), 0.1% SDS) for at least 1 h at 60°C.

Catenated pRS316 plasmids or replication intermediates from pRS426-RFB were probed with DNA amplified from pRS316 (probing specifically for the *URA3* gene). Labelling and detection used random prime labelling incorporating fluorescein tagged dUTP (Roche). Following probing, hybridized fluorescein tagged dUTP was detected with alkaline phosphatase tagged anti fluorescein Fab fragments (Roche), revealed with CDP-Star (GE Healthcare) and non-saturating exposures acquired on an ImageQuant LAS4000 system (GE Healthcare). Densitometry analysis was carried out using ImageQuant TL software.

### FACS analysis of DNA content

For analysis of cell cycle progression, 0.5 ml of yeast culture was pelleted by centrifugation at 13 000 rpm for 15 s before removal of all growth media. The pellets were re-suspended in 0.5 ml 70% ethanol and stored at 4°C before processing and analysis.

Fixed cells were washed in 50 mM Tris pH 8.0 and 10 mg/ml RNaseA (Sigma-Aldrich) was added. Cells were incubated overnight at 37°C, pelleted and re-suspended in freshly made 5 mg/ml pepsin (Sigma) in 5 mM HCl and incubated again at 37°C for 30 min. Fixed cells were washed once more in 50 mM Tris pH 8.0 and re-suspended in 0.5 mg/ml propidium iodide in 50 mM Tris pH 8.0. Samples were sonicated for 5 s each on low power to reduce clumping before analysis using the BD Accuri™ C6 Plus (BD Biosciences).

### Drug sensitivity assays

Yeast cells were grown to mid-log phase before being serially diluted 10-fold in YPD. 5 μl of each dilution was spotted onto YPD plates containing the indicated dose of drug or control reagent and incubated for 48 h at 25°C or 30°C before imaging.

### Colony survival assays

Yeast cells were grown to mid-log phase before being arrested in G1 by the addition of 10 μg/ml alpha factor peptide. When cells were >90% unbudded they were released into the cell cycle in the presence of 200 mM HU for 1 h. Following the HU treatment cells were counted, diluted in YPD medium and plated onto YPD plates. Colonies were counted 48 h after plating and the viability was calculated as the percentage of plated cells able to form colonies. Statistical significance was calculated using a two-tailed unpaired Students t-test.

### RPA1 ChIP-seq

Pellets from 75 ml cultures were resuspended in 500 μl SDS buffer (1% SDS, 10 mM EDTA, 5M Tris HCl, cOmplete Tablets Mini EDTA-free EASYpack (Roche), PhosSTOP (Roche)). 200 μl of 0.5 mm zirconia/silica beads were added to samples and cells were lysed using the FastPrep-24 (MP Biomedicals) on max speed (6.5 m/s), with five rounds of 1 min each. Lysate was spun out and IP buffer (0.1% SDS, 1.1% Triton-X-100, 1.2 mM EDTA, 16.7 mM Tris–HCl (pH8), cOmplete Tablets Mini EDTA-free EASYpack (Roche), PhosSTOP (Roche)) was added to a final volume of 1 ml. Samples were sonicated using the Focused-Ultrasonicator (Covaris) (average incident power 7.5 W, peak incident power 75 W, duty factor 10%, cycles/burst 200, duration 20 min). The sample was centrifuged for 20 min at 13 000 rpm at 4°C. Supernatant was then diluted to 7.5 ml with IP buffer. 75 μl protein A Dynabeads (Invitrogen) and 75 μl protein G Dynabeads (Invitrogen), were washed 3 times in IP buffer before adding to the sample and incubating for 2 h at 4°C. 2 ml of the supernatant was taken to 15 ml falcon tubes, and the rest was kept at −20°C as an input sample. To the 2 ml sample RPA1 antibody (1:10 000, Agrisera, AS07214) was added followed by overnight incubation on a rotating wheel at 4°C.

A mix of Dynabeads, Protein A (30 μl) and Protein G (30 μl), was washed 3 times in IP buffer. This was added to each sample and incubated at 4°C for 4 h. Supernatant was removed and beads were washed at 4°C for 6 min in TSE-150 (1% Triton-X-100, 0.1% SDS, 2 mM EDTA, 20 mM Tris–HCl (pH8), 150 mM NaCl), followed by TSE-500 (1% Triton-X-100, 0.1% SDS, 2 mM EDTA, 20 mM Tris–HCl (pH8), 500 mM NaCl), followed by LiCl wash (0.25 M LiCl, 1% NP-40, 1% dioxycholate, 1 mM EDTA, 10 mM Tris–HCl (pH8)) and finally Tris-EDTA (TE pH8). Elution was carried out in 400 μl elution buffer (1% SDS, 0.1M NaHCO_3_) for 30 min at room temperature. At the same time 50 μl from the input sample was added to 150 μl of elution buffer. 20 μl of 5 M NaCl and 10 μl of 10 mg/ml proteinase K (Invitrogen) was then added to the input, and 40 μl and 20 μl to the IP samples respectively. These were incubated at 65°C overnight. Then 10 μl of DNase-free RNase (Roche) was added to the input and 20 μl to the IP samples, and they were left at 37°C for 30 min. All DNA was purified with a Qiagen PCR purification kit and eluted in 40 μl H_2_O. 34 μl from the RPA1 samples and 1 ng DNA in 34 μl water from the input were used for library preparation. 5 μl 10× NEB2.1 buffer and 5 μl of random primers (8N, 3 mg/ml stock) were added and the samples were boiled at 95°C for 5 min and immediately placed on ice for 5 min. 5 μl 10× dNTPs with dUTP instead of dTTP (2 mM each) and 1 μl T4 polymerase (NEB) were added and the mixture was incubated at 37°C in a thermal cycler for 20 min, and 5 μl 0.5 M EDTA (pH 8) was immediately added to stop the reaction. The resulting dsDNA was used to create libraries using the Ultra II library kit (NEB) as per the manufacturer's instructions with 13 cycles at the amplification step.

Paired end sequencing was performed using the MySeq (75 bp reads from each side) or NextSeq 500 (42 bp reads from each side) systems to result >2 million reads.

### ChIP-SEQ analysis

FASTQ files were generated by Illumina basespace (https://basespace.illumina.com/home/index). The resulting sequences were aligned to a reference genome (R64–1-1, *Saccharomyces cerevisiae* S288c assembly from Saccharomyces Genome Database) using Bowtie 2 generating a SAM output file for each sample (http://bowtie-bio.sourceforge.net/bowtie2/index.shtml). Reads from MySeq were trimmed 25 bp from 3′ and 1 bp from the 5′ end, while reads from NextSeq were not trimmed.

Command for MySeq reads:

bowtie2 -p 14 -x [path to index folder] –trim3 25 –trim5 1 -1 [Path and name of R1 fastq file] -2 [Path and name of R2 fastq file] -S [name of the resulting .sam file]

Command for NextSeq reads:

bowtie2 -p 14 -x [path to index folder] –trim3 0 –trim5 0 -1 [Path and name of R1 fastq file] -2 [Path and name of R2 fastq file] -S [name of the resulting .sam file]

SAM files were then converted into sorted BAM files by using SAMtools (http://samtools.sourceforge.net/):

samtools sort [name of the .sam file generated with bowtie2] -o [name for the resulting .bam file] -O bam -T [name for resulting .bam file wo .bam]

Duplicates were then removed using picard (https://broadinstitute.github.io/picard)

java -jar ∼/picard/picard-tools-1.138/picard.jar MarkDuplicates I = [name for the resulting .bam file] O = [name for the resulting without repeats.bam file] M = [name of metrix file.txt] REMOVE_DUPLICATES = true

BAM files were used for Model-based Analysis of ChIP-Seq (MACS2). We used the ‘call peak’ function which also generates genome wide score data. These were used to generate fold enrichment tracks. Example command:

macs2 callpeak -t [sorted BAM file from yh2a data]-c [sorted BAM file from h2a data]-f BAMPE -g 12100000 -n [name for output file] -B -q 0.01 –SPMR

The data then was sorted into 50 bp bins, normalized to have a mean value of 1, and used for meta data analysis using custom-made R programs.

Regions within 10 kb of LTR-retrotransposons, transposable element genes, rRNA genes and long terminal repeats were removed from the meta analysis as these regions have high RPA enrichment.

### Sync-seq

Pellets from 2 ml cultures were resuspended in 500 μl SDS buffer (1% SDS, 10 mM EDTA, 5 M Tris–HCl, cOmplete Tablets Mini EDTA-free EASYpack (Roche), PhosSTOP (Roche)). 200 μl of 0.5 mm zirconia/silica beads were added to samples and cells were lysed using the FastPrep-24 (MP Biomedicals) on max speed (6.5 m/s), with five rounds of 1 min each. Lysate was spun out and IP buffer (0.1% SDS, 1.1% Triton-X-100, 1.2 mM EDTA, 16.7 mM Tris–HCl (pH8), cOmplete Tablets Mini EDTA-free EASYpack (Roche), PhosSTOP (Roche)) was added to a final volume of 1 ml. Samples were sonicated using the Focused-Ultrasonicator (Covaris) (average incident power 7.5 W, peak incident power 75 W, duty factor 10%, cycles/burst 200, duration 20 min for G1 and HU released samples and 13.5 for HU samples). 200 μl of sample was removed and 10 μl of DNase-free RNase was added and incubated at 37°C for 30 min. DNA was then purified with a Qiagen PCR purification kit and eluted in 50 μl H_2_O. 50 ng of DNA in 50 ul water was used for library preparation using the Ultra II library kit (NEB) as per the manufacturer's instructions with 6 cycles at the amplification step.

Paired end sequencing was performed using NextSeq 500 (42 bp reads from each side) systems to result >2 million reads.

### Sync-seq analysis

Sync-seq analysis (29) was carried out using LocalMapper shell script and Repliscope R package from https://github.com/DNAReplicationLab/.

localMapper.sh -g [path to index folder] [Path and name of R1 fastq file] -2 [Path and name of R2 fastq file] -s [name of the output files] -w 3000 -c 14

The resulting .bed files were then read in to R:

repBed ← loadBed(file name for the replicating sample)
nrepBed ← loadBed(file name for the non-replicating sample)

Outliers were removed:

repBed←rmOutliers(repBed, ‘median’, loLim = 0.25)
repBed←rmOutliers(repBed, ‘max’, n = 2)
nrepBed←rmOutliers(nrepBed, ‘median’, loLim = 0.25)
nrepBed←rmOutliers(nrepBed, ‘max’, n = 2)

Ratio between non-replicated and replicated samples were calculated:

ratio ← makeRatio(repBed,nrepBed)

And normalised:

ratio ← normaliseRatio(ratio, [rFactor])

where rFactor was empirically determined to fit the lowest replicating regions to 1.

The resulting ratios were smoothed by a moving average of 7 and plotted using custom-made R programs. 

## RESULTS

### Tof1 mutants lacking Top1 binding capability cannot suppress replication fork hyper-rotation

**Figure 1.**
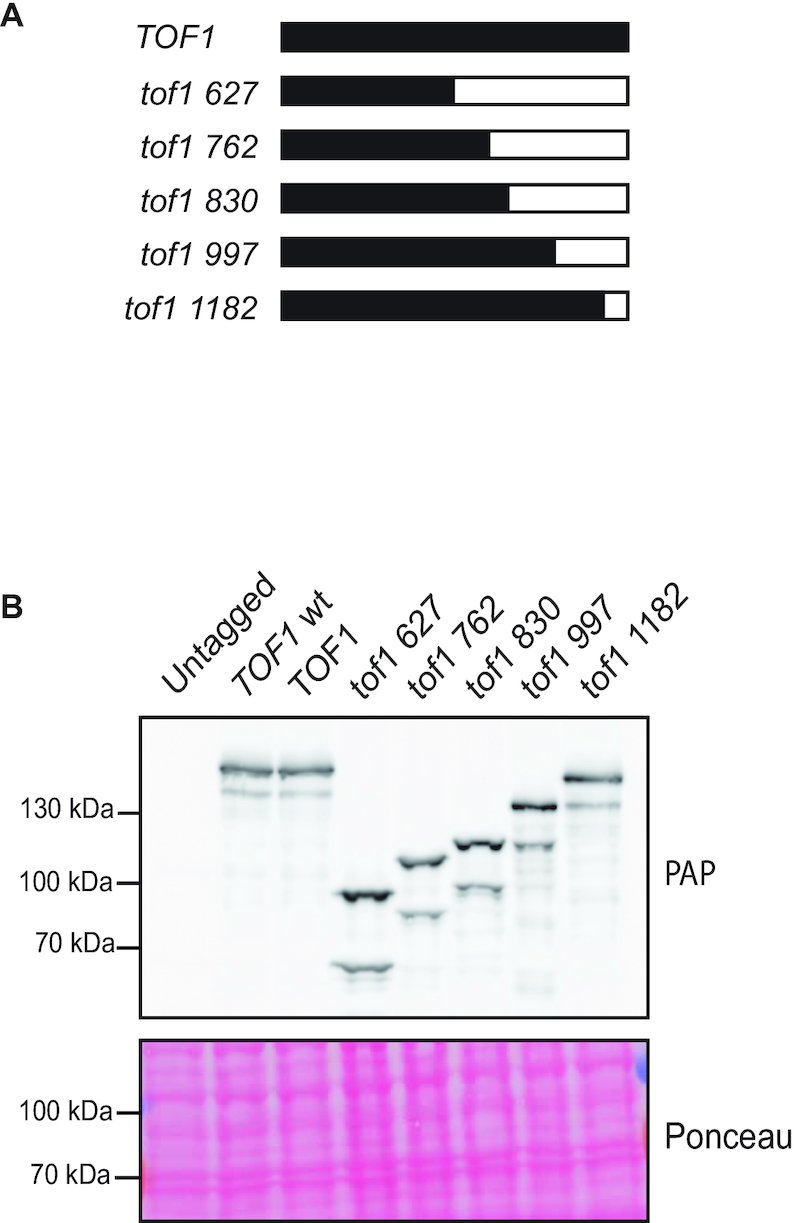
Expression of C terminal Tof1 truncation mutations. (**A**) Schematic showing length of Tof1 protein expressed by each of the C terminal truncation mutations compared to wildtype Tof1 (1238 aa). (**B**) Western blot showing expression levels of C terminal Tof1 truncation mutants. Endogenous TOF1 (*TOF1wt*), codon-optimised *TOF1* (*TOF1*) and *tof1* mutants were C terminally tagged with TAP epitope and lysates were checked for expression using peroxidase anti-peroxidase (PAP) which immuno-reacts with the protein A portion of TAP tags. Ponceau stain of blotted membrane is shown to illustrate protein content of lanes. A representative western of the two experiments performed is shown.

In order to dissect which domains of the 1238 amino acid (aa) long Tof1 protein are required for each of its characterised functions we generated a series of premature stop codons in a *TOF1* ORF sequence optimised for budding yeast expression (12). Premature stop codons were introduced at aa 627, aa 762, aa 830, aa 997 and aa 1182 (Figure 1A) and the truncated alleles expressed from the endogenous *TOF1* locus. C terminal tagging of each of these alleles demonstrated that they generated proteins of the predicted full-length size, which were expressed to levels comparable to that expressed with exogenous codon-optimised *TOF1* and endogenous *TOF1* (Figure 1B). We noted that both *TOF1* and mutated *tof1 C* terminally tagged alleles also generated a faster mobility form of protein, in addition to the predicted full-length protein (Figure 1B). The size difference between these two forms appeared to be the same for both the full-length and truncation alleles, suggesting that the difference was due to a modification in the N terminal half of the protein. To investigate this further we tagged the N terminus of *TOF1, tof1 627, tof1 997* and *tof1 1182* with the HA epitope and re-examined expression (Supplementary Figure S1). We only observed the full-length protein in this case, indicating that the faster mobility form of Tof1 lacks the beginning of the N terminus of the full-length protein. Since both the full length and N terminal variant of the wildtype and truncated proteins are altered by the same C terminal truncations, we continued with our analysis of the requirement of C terminal regions for function.

**Figure 2.**
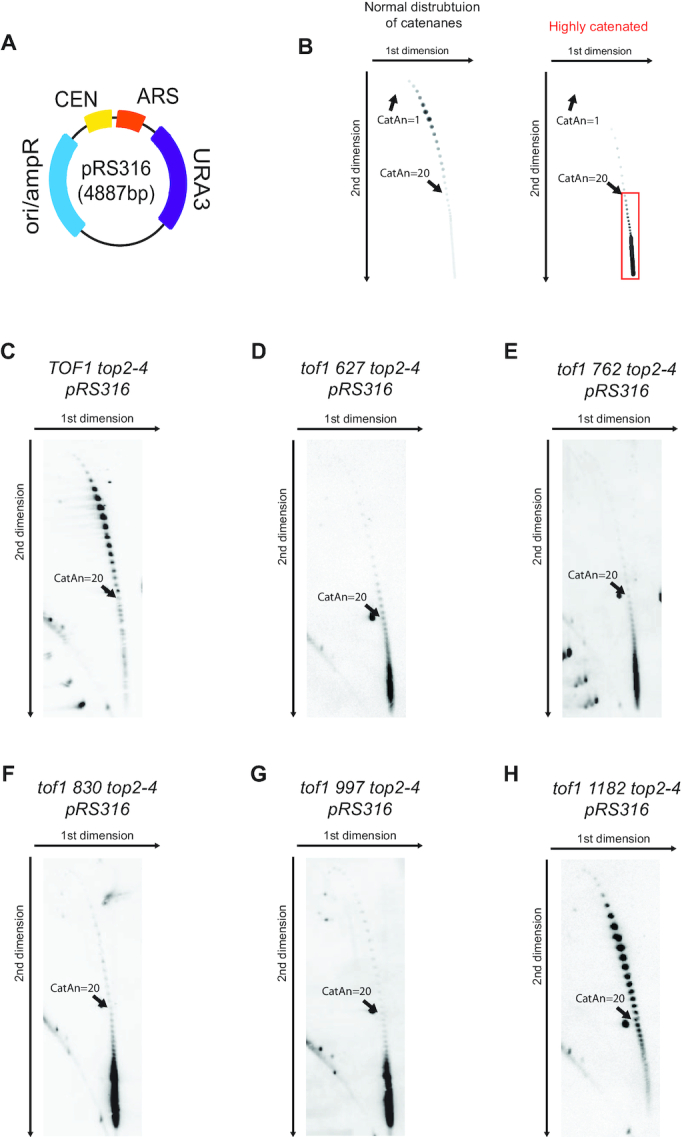
Suppression of hyper-catenation on episomal plasmids in Top2-inactivated cells requires the far C terminal region of Tof1. (**A**) Plasmid pRS316 used for DNA catenation analysis. (**B**) Schematic showing the representative increase in DNA catenation of plasmid pRS316 in *tof1Δ top2–4* cells compared to *top2–4* cells as visualized by Southern blotting. Region of hypercatenated plasmids highlighted in red box. (**C-H**) DNA catenation analysis of pRS316 in *top2–4* cells expressing (**C**) *TOF1* (**D**) *tof1 627* (**E**) *tof1762* (**F**) *tof1830* (**G**) *tof1997* (**H**) *tof1 1182* Images shown are from one of two equivalent independently conducted experiments.

To understand how truncation of the C terminus affects Tof1 function, we first set out to establish which of the truncated Tof1 proteins could suppress the excessive fork rotation phenotype of *tof1Δ* cells, as visualised by Southern blotting of replication products (22). In cells with wildtype function of *TOF1*, episomal plasmids accumulate only modest levels of DNA catenanes during S phase following Top2 inactivation (Figure 2A). This demonstrates that fork rotation is relatively infrequent and therefore that DNA topological stress is primarily resolved by Top1 ahead of the replication fork in this context (22). In contrast, *tof1Δ* cells accumulate hyper-catenated plasmids during S phase indicating that, in cells lacking Tof1, DNA topological stress is resolved far more frequently by fork rotation and action of topoisomerase behind the fork (Figure 2B). To assess whether the truncated proteins were capable of suppressing hyper-catenation and thus restoring the primacy of Top1 action ahead of the fork, we replaced *TOF1* with each of the *tof1* truncation alleles encoding the truncated proteins into *top2–4* cells containing the plasmid pRS316. Following synchronisation in G1, we cultured the cells at the restrictive temperature to ablate Top2 activity and released the cells into the cell cycle for one passage through S phase. We then harvested the cells prior to mitosis, preventing further cell cycle progression with the microtubule depolymerising drug nocodazole. We extracted DNA and assessed the frequency of DNA catenation introduced into the plasmid using two-dimensional gel electrophoresis and Southern blotting. As expected, expression of *TOF1* completely suppressed excessive fork rotation and DNA catenation (Figure 2C). Expression of *tof1 1182* which lacks only the final 57 amino acids of the C terminus of Tof1 also fully rescued the excessive fork rotation (Figure 2H). However, expression of Tof1 proteins truncated at 627, 762, 830 and 997 did not rescue this effect (Figure 2D-G). This indicates that the region of Tof1 between aa 997 and aa 1182 is required to suppress excessive fork rotation. This region is known to be capable of supporting a two-hybrid interaction with Top1 (23) and is required to recruit Top1 to the replication fork (24). To certify that the interaction between Tof1 and Top1 requires the region between aa 997 and aa 1182, we pulled down C terminally TAP tagged *tof1* truncation proteins and probed for interaction with myc tagged Top1 protein. This analysis confirmed that TAP tagged *tof1 1182* protein could pull down myc tagged Top1 in cells (Supplementary Figure S2). In contrast, TAP tagged *tof1* 627 and 997 proteins did not (Supplementary Figure S2). This is consistent with the model that preferential recruitment of Top1 to the replisome stimulates its action ahead of the fork (3,22).

### The fork pausing function of Tof1 is separate from its role in topological stress

**Figure 3.**
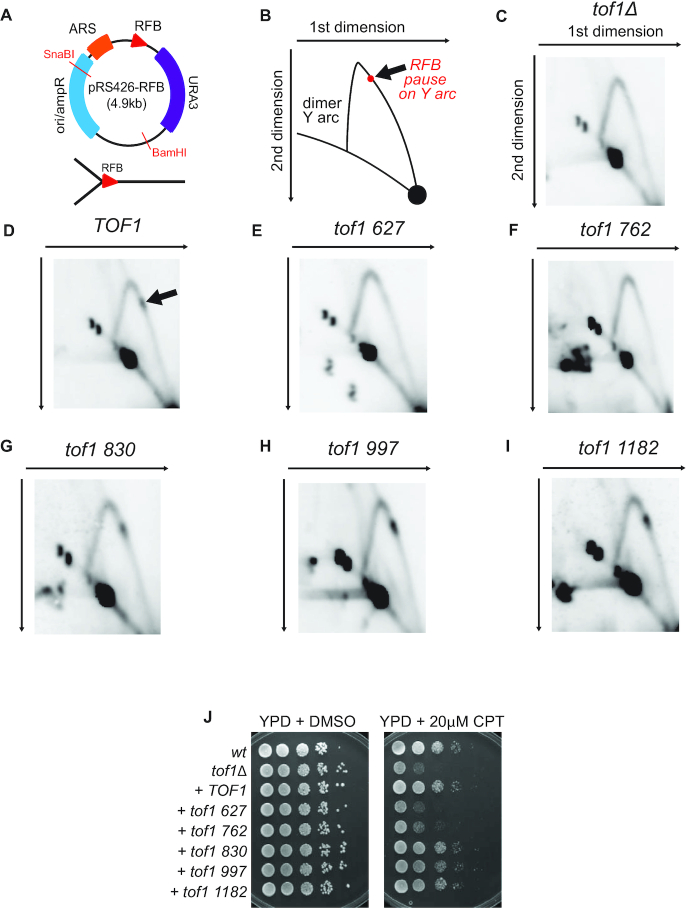
Recovery of fork pausing and resistance to camptothecin requires the central region of Tof1 but not the far C terminal region. (**A**) Plasmid pRS426-RFB used for analysis of fork pausing at the RFB. The two unique restriction digest sites used to linearize the plasmid for 2D replication fork gel analysis and the Y structure anticipated to be generated by fork pausing at the RFB pause site are shown. (**B**) Schematic of the replication fork Y arc predicted to be generated by pRS426-RFB digestion with BamHI and SnaBI in wildtype cells and resolved by 2D gel electrophoresis. Arrow indicates the accumulation of replication intermediates generated by pausing at the RFB on pRS426-RFB. (**C–I**) 2D gel analysis of BamHI and SnaBI digested pRS426-RFB extracted from exponentially growing cells containing (**C**) *tof1Δ* (**D**) *TOF1* (**E**) *tof1 627* (**F**) *tof1762* (**G**) *tof1830* (**H**) *tof1997* (**I**) *tof11182* (**J**) Viability spot assay of wildtype (*wt*), *tof1Δ, TOF1, tof1 627, tof1 762, tof1 830*, *tof1 997*, and *tof1 1182* cells. Cells were spotted in ten-fold sequential dilutions on either YPD + DMSO or YPD plus 20 μM camptothecin (CPT) plates and incubated for 48 h at 25°C. Images shown are from one of two equivalent independently conducted experiments.

We next set out to determine which of the mutants were able to support replication fork pausing at a replication block. We cloned the *S. cerevisiae* rDNA region that contains the replication fork barrier (RFB) (corresponding to Chromosome XII sequence: 459799–460920) into the multicopy yeast episomal plasmid pRS426 (Figure 3A). Transplanting the RFB sequence into an artificial location pauses replication forks in a Fob1 dependent manner but does not fully arrest ongoing replication (20). It therefore appears to act in a similar manner to other endogenous protein pausing sites (21). We used both a BamHI/SnaBI double digestion of plasmid pRS426-RFB extracted from yeast cells to examine Y arcs containing the RFB (cartoon of replication intermediates revealed shown in Figure 3B) (Figure 3C–I) or SnaBI single digestion to examine the replication intermediates of the entire replicon (Supplementary Figure S3) (30). We found that cells without Tof1 did not pause at the RFB site on the plasmid (Figure 3C, Supplementary Figure S3B), whereas cells expressing *TOF1* fully supported pausing at this location (Figure 3D, Supplementary Figure S3C). Expression of *tof1 830, tof1 997 and tof1 1182* also fully supported pausing (Figure 3G–I, Supplementary Figure S3F-H). However, expression of *tof1 627* and *tof1 762* did not rescue pausing at the RFB site (Figures 3E and F, Supplementary Figure S3D and E). Together this data indicates that the region beyond aa 830 is not required for replication fork pausing at protein barriers and therefore that the role of Tof1 in replication fork pausing is independent of its role in inhibiting fork rotation.

Deletion of *TOF1* results in hypersensitivity to CPT, an agent which stabilises the Topoisomerase 1 covalent complex (Top1cc) to DNA, forming a DNA protein cross-link (DPC). Deletion of *CSM3* results in similar sensitivity to CPT treatment while *mrc1*Δ cells display only mild sensitivity to this agent (25). Therefore, CPT sensitivity appears to be a marker for functions of the Tof1–Csm3 heterodimer that are independent of their association with Mrc1. The cause of the acute toxicity of *tof1Δ* or *csm3Δ* cells to CPT is currently unclear. On the leading strand a DPC and an adjacent single strand DNA break would be predicted to either inhibit replication fork progression or generate a double strand break (31,32). Alternatively, CPT treatment has also been found to cause increased DNA topological stress on cellular DNA (33), potentially generating a situation where efficient recruitment of topoisomerases to the fork by Tof1 is required for ongoing elongation. Our data shows that *tof1* truncations that cannot support relaxation of DNA topological stress through Top1 recruitment can still support fork pausing at an RFB complex. We therefore examined how expression of each of the truncation mutations suppressed the sensitivity of *tof1Δ* cells to CPT. Cells expressing *tof1 627* or *tof1 762* were highly sensitive to CPT while expression of *tof1 830, tof1 997* and *1182* provided *wt* levels of resistance to CPT (Figure 3J). Therefore, truncation mutants that do not support Tof1’s role in promoting Top1 action ahead of the fork (*tof1 830* and *tof1 997*) have normal levels of resistance to CPT. Conversely, *tof1* truncations that do not support replication fork pausing (*tof1 627* and *tof1 762*) cannot suppress CPT sensitivity. We conclude that CPT sensitivity is closely associated with the ability of the Tof1-Csm3 complex to support pausing of the fork at a protein block to replication.

Despite our findings that the C terminal region beyond aa 830 is not required for CPT resistance, we also observed that some mutations located C terminal of aa 830 can still impair cellular resistance to CPT. While assessing the function of conserved domains in the C terminus we found that an internal deletion of the region coding for aa 1050–1080 results in impaired CPT resistance (Supplementary Figure S4A). We conclude that although the C terminal region beyond aa 830 is not required for CPT resistance and fork pausing, disruption of the polypeptide structure of this region can disrupt the function of other regions of the protein. Such a characteristic of the C terminus could explain why another study has found that a Tof1 protein truncated at aa 981 by addition of a FLAG peptide tag causes CPT sensitivity and reduced pausing at the RFB in cells (24). Potentially the addition of a peptide tag could induce a distortion in the C terminal region capable of disrupting activity elsewhere in the protein. We therefore tested the possibility that a C terminal tag in this region could deleteriously affect Tof1 function (Supplementary Figure S4B). We observed that addition of a C terminal TAP tag to the tof1 997 protein causes a clear increase in the CPT sensitivity of *tof1 997* expressing cells. Addition of a C terminal TAP tag did not adversely affect all regions of the protein, as the tagged forms of *TOF1* or *tof1 627* did not appear to be more sensitive to CPT than their untagged forms (Supplementary Figure S4B). Our data argues that while the polypeptide structure of the first 830 aa is sufficient for fork pausing and CPT resistance functions of Tof1, structural deformation in the C terminal region beyond this point can still alter the activity of the rest of the protein.

### The replisome coupling function of Tof1 is associated with its role in fork pausing

**Figure 4.**
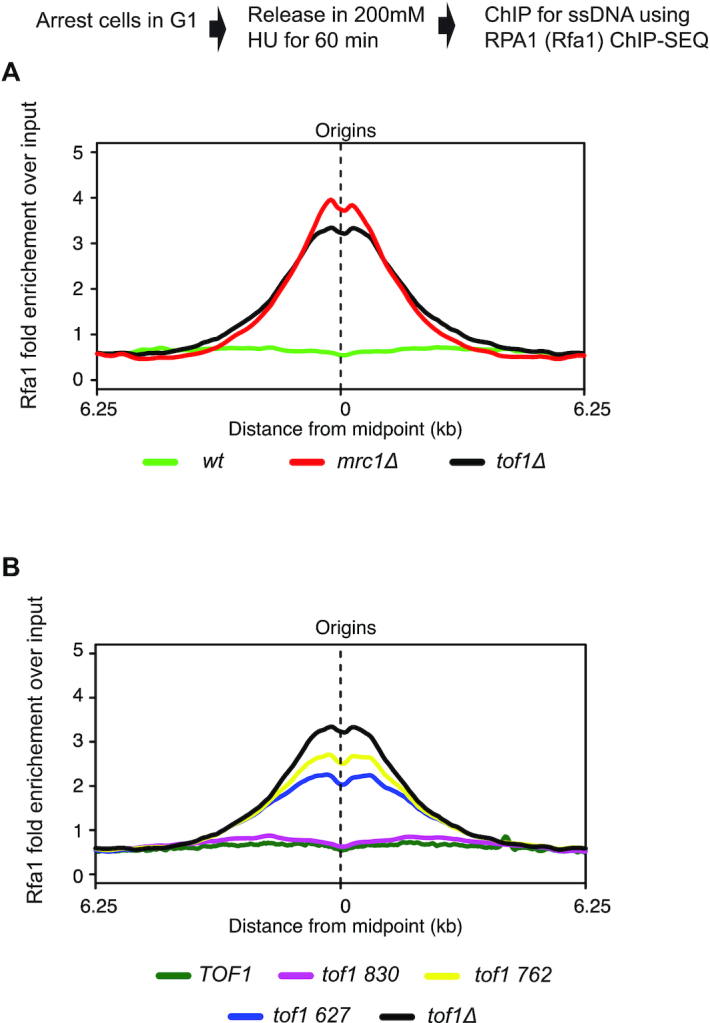
Coupling of helicase and polymerase activities requires the central region of Tof1 but not the far C terminal region. To examine the extent of fork uncoupling we assayed ChIP of RPA1 (*Sc*Rfa1) following 200 mM hydroxyurea (HU) arrest of cell proliferation. (**A**) Schematic of experimental set up and meta-analysis of enrichment of RPA1 (*Sc*Rfa1) around origins in *wt, tof1Δ* and *mrc1Δ* cells following release of G1 cells into media containing 200 mM HU for 60 min. (**B**) Meta-analysis of enrichment of RPA1 (*Sc*Rfa1) around origins in *TOF1, tof1Δ tof1 627, tof1 762* and *tof1 830* cells following release of G1 cells into media containing 200 mM HU for 60 min. Data shown is from merging two independently conducted Rfa1 ChIP-SEQ experiments in each background.

Replisome pausing induced by nucleotide depletion with hydroxyurea (HU) also requires Tof1 function. Following acute treatment of cells with HU both Tof1 and Mrc1 are required to maintain the coupling of helicase and polymerase activities at the fork (15). In the absence of Tof1 or Mrc1 the helicase advances beyond the point of nascent DNA incorporation, indicating uncoupling of helicase and polymerase activities in the replisome (15). This uncoupling is predicted to lead to increased binding of the single stranded DNA-binding protein, RPA1, to the single stranded regions generated by uncoupled helicase action. We have experimentally confirmed this prediction using RPA1 ChIP-SEQ in HU treated wildtype, *tof1Δ* and *mrc1Δ* cells. Release of cells arrested in G1 with alpha factor into media containing 200 mM HU led to strongly increased RPA1 chromatin binding around replication origins in *tof1*Δ and *mrc1*Δ cells compared to wildtype (Figure 4A). Expression of *TOF1* and *tof1 830* both suppressed the accumulation of RPA1 around origins (Figure 4B). Thus, expression of the *tof1 830* truncation mutant was sufficient to ensure helicase and polymerase coupling. However, expression of either *tof1 762* or *tof1 627* still led to increased RPA1 around origins, indicating uncoupling of helicase and polymerase activities. Notably the elevated level of RPA1 around origins in either *tof1 762* or *tof1 627* was less than the level of RPA1 observed in *tof1*Δ cells (Figure 4B), indicating that they retained some function in coupling of helicase and polymerase activities, potentially through their mediation activity in the DRC (see below).

### The C terminus of Tof1 beyond aa 627 is dispensable for DRC activation and replication fork restart but required for cell viability following fork restart

**Figure 5.**
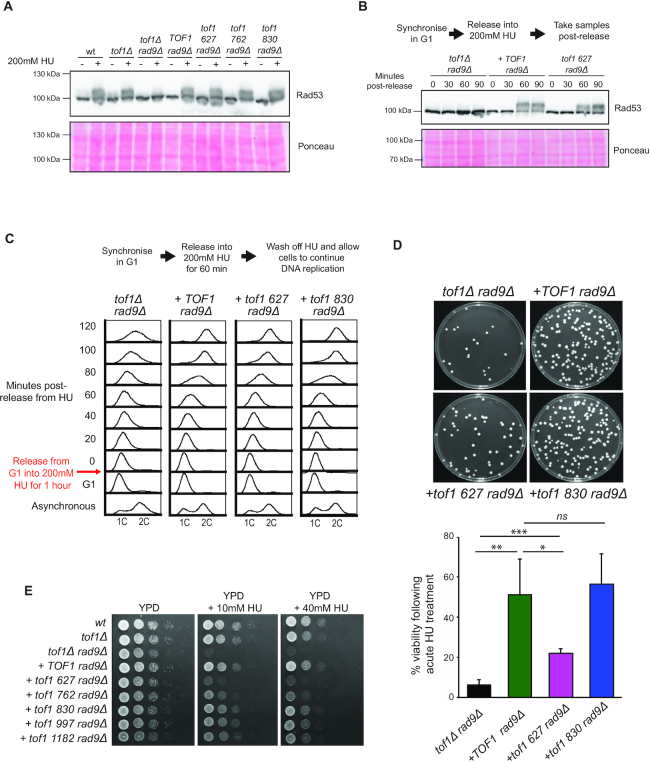
Following HU arrest the N terminal half of Tof1 is sufficient for DRC activation and resumption of DNA synthesis but not cell viability in DDC deficient (*rad9Δ*) cells. (**A**) Rad53 activation assay following HU treatment. Activation of Rad53 causes autophosphorylation that results in reduced mobility of the Rad53 protein. The relative mobility of Rad53 in each of the indicated cell types either in exponentially growing cells or following a 2 h treatment with 200 mM HU was assayed by western blot using anti-Rad53 antibodies (top). Ponceau stained membrane of western blot shown in top panel indicates relative protein levels (bottom). Image shown is from one of two equivalent independently conducted experiments. (**B**) (Top) Time course of Rad53 activation following release from G1 into media containing 200 mM HU. (Bottom) Ponceau stained membrane of western blot shown in top panel indicates relative protein levels. Image shown is from one of two equivalent independently conducted experiments. (**C**) Recovery of DNA synthesis in cells following cell cycle arrest induced by 200 mM HU. The indicated cell mutants were arrested in 200 mM HU following release of a G1 synchronised culture. After 60 min in HU, the HU containing media was washed off and cells allowed to recover and resume passage through S phase. Shown are FACS profiles of DNA content before and after the HU arrest point. Time points indicate the time in minutes after release from the HU arrest (T0 indicates time from the first wash). Images of DNA content FACS shown are from one of two equivalent independently conducted experiments. (**D**) Viability of cells following cell cycle arrest induced by acute HU treatment. G1-arrested cells were released from the block into YPD media containing 200 mM HU for 60 min, after which the cultures were diluted and plated to YPD plates. Plates were incubated for 48 h at 25°C before images were taken (top). Colony number was counted 48 h after plating and results from 4 repeats were quantified and displayed in histogram (bottom). *P* values: * = <0.05, ** = <0.01, *** = <0.001 calculated using a two-tailed unpaired Student's *t*-test using four repeats for each mutant tested. (**E**) Viability of cells during chronic treatment with HU. Viability spot assay of *TOF1*, *tof1Δ, tof1 627, tof1 762, tof1 830*, *tof1 997* and *tof1 1182* cells on YPD or YPD plus 10 or 40 mM HU for three days at 25°C. Images shown are from one of two equivalent independently conducted experiments.

We next set out to establish whether expression of the truncated proteins was sufficient to support the mediator function of Tof1 in activation of the DRC. In *S. cerevisiae* sustained activation of the effector kinase Rad53 in S phase following HU treatment can either be through the DRC, mediated by Mrc1 and Tof1, or through the Rad9-mediated DDC (8). Therefore, loss of detectable activation of Rad53 in response to HU only occurs in cells lacking both *tof1* and *rad9* function. Conversely, HU-dependent Rad53 activation can be rescued by the activity of either Tof1 or Rad9 protein in *tof1Δ rad9Δ* cells. We assayed whether expression of any of the *tof1* truncation mutants could rescue Rad53 activation in response to HU treatment in *tof1Δ rad9Δ* cells, using the Rad53 phospho-mobility assay (34). In these experiments, treatment of exponentially growing *wt* cells with 200 mM HU led to a robust auto-phosphorylation-linked mobility shift of Rad53 protein as visualised by western blot. This shift was partially attenuated in *tof1Δ* and lost in *tof1Δ rad9Δ* cells (Figure 5A). Interestingly, expression of all of the truncation mutants rescued the phosphorylation-linked mobility shift of Rad53 in response to HU (Figure 5A). Therefore, the first 627 aa of Tof1 retains the ability to act as a mediator for the DRC. Previous studies have shown that hypo-morphic alleles of some checkpoint components delay but do not prevent the full activation of Rad53 following treatment with HU (35). To assess whether this was the case for the *tof1 627* allele we assayed protein extracts from cells taken at sequential time points following release into 200 mM HU for Rad53 activation (Figure 5B). We did not observe a delay in activation of Rad53 in the *tof1 627* allele compared to wildtype Tof1, consistent with this allele activating the intra-S-checkpoint with wildtype kinetics.

The separation of function observed between the roles of Tof1 in checkpoint signalling and in replisome coupling/pausing provided us with an opportunity to differentiate between the contributions of these two Tof1 functions to genome stability following RS induced replisome stalling. Following treatment of budding yeast with 200 mM HU, replication forks stall shortly after origin firing, activating the DRC. DRC activation is essential to maintain cellular viability during incubation in HU (36,37). Checkpoint-dependent viability is thought to be due to regulation of several pathways including inhibition of late origin firing (38), inhibition of nucleases that could process the arrested fork (39,40) and stabilisation of replisome components at the arrested fork (36,37). The role of Tof1 in maintaining genome stability in response to RS could either be through mediation of checkpoint signalling or through its pausing/coupling activities aiding maintenance of replisome integrity, or a combination of the two. To investigate the contribution of these two roles to genome stability we compared the response to arrest and subsequent wash off of 200 mM HU in *tof1*Δ *rad9*Δ cells, where the checkpoint activation of Rad53 is ablated, to *tof1*Δ *rad9*Δ cells complemented with either *TOF1*, *tof1 627* or *tof1 830*, all of which generate Tof1 protein capable of activating the DRC in *tof1*Δ *rad9*Δ cells (Figure 5C and 5D). Using FACS analysis of DNA content, we observed that uncomplemented *tof1*Δ *rad9*Δ cells were unable to complete DNA replication following removal of 200 mM HU, consistent with previous analysis of checkpoint-defective cells (36,41) (Figure 5C). Complementation of these cells with wildtype Tof1 resulted in rapid completion of DNA replication (Figure 5C). In addition, complementation of *tof1*Δ *rad9*Δ cells with either of the checkpoint competent truncation mutants *tof1 627* or *tof1 830* also resulted in rapid apparent completion of DNA replication (Figure 5C), consistent with the checkpoint function of Tof1 alone being sufficient for resumption of DNA replication following HU arrest. In addition to testing DNA content we also re-plated these cells following release from the acute HU treatment onto YPD plates to assess whether they could survive the treatment. As expected, *tof1*Δ *rad9*Δ cells failed to form colonies following acute HU treatment whereas *TOF1 rad9Δ* cells displayed efficient colony-forming capability (Figure 5D). Despite their apparent resumption in DNA replication following acute HU treatment, *tof1 627* cells generally failed to recover from the HU-induced arrest, although they were significantly more viable than *tof1*Δ *rad9*Δ cells (Figure 5D). This indicates that despite DRC activation and apparent efficient resumption of DNA replication, *tof1 627* expressing cells were still subject to frequent unrecoverable lesions following fork stalling. Consistent with this interpretation both *tof1 627* and *tof1 762* cells were acutely sensitive to chronic treatment with HU compared to the other *tof1* truncation mutants in *rad9*Δ cells (Figure 5E). This indicates that Tof1 has roles in maintaining cellular viability during replication stress beyond activation of the DRC and resumption of DNA replication.

**Figure 6.**
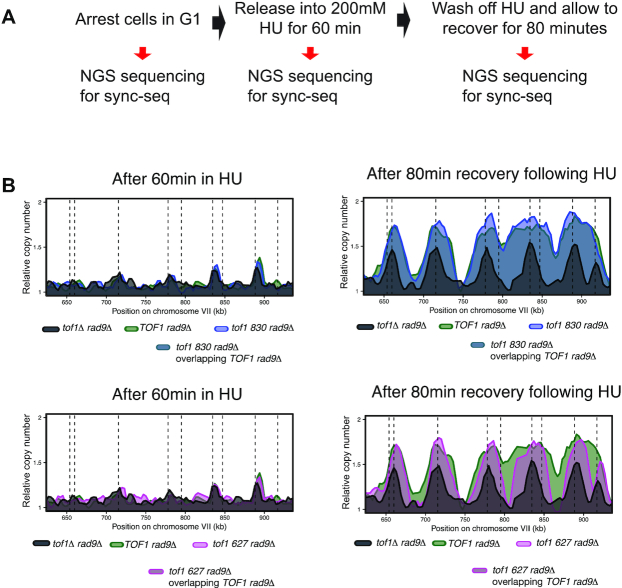
Replication in *tof1 627* cells is reduced after release from HU treatment in *rad9Δ* background. (**A**) Experimental set up for sync-seq experiments in cells arrested in 200 mM HU and released to follow replication fork restart. (**B**) Overlaid relative copy number of *TOF1 rad9Δ* and *tof1Δ rad9Δ* compared to *tof1 830 rad9Δ* (top panels) and *tof1 627* (lower panels) after 60 min exposure to 200 mM HU (left panels) and 80 min after wash off of HU allowing the cells to recover and resume DNA replication (right panels). Dashed lines represent known origins. Data from one of two independent sync-seq experiments is shown as representative of both replicates.

FACS analysis of DNA content cannot distinguish if any increases in DNA content are due to restart of arrested replication forks or due to new origins firing. To investigate this issue, we carried out sync-seq analysis (41,42) to compare copy number differences across the genome between cells arrested in 200 mM HU and cells 80 minutes following wash off of HU, recovery and resumption of DNA replication (Figure 6A). Analysis of *TOF1 rad9Δ*, *tof1 627 rad9Δ*, *tof1 830 rad9Δ* and *tof1Δ rad9Δ* cells arrested in HU all showed similar copy number in the immediate vicinity of early firing origins as expected (Figure 6B left). Further sync-seq analysis of the same cultures 80 minutes after HU removal showed that *TOF1* *rad9*Δ and *tof1 830* *rad9*Δ cells had completed replication around origins fired before HU arrest as anticipated, indicating that stalled forks in these cells were able to restart replication following release from the HU block (Figure 6B top right). As predicted, *tof1Δ rad9Δ* cells had a much smaller increase in copy number around origins consistent with poor levels of recovery or restart from HU-arrested forks in this genetic background (Figure 6B right). In contrast to these two extremes, *tof1 627* cells exhibited a similar pattern of increased copy number around origins fired in HU to *wt* and *tof1 830*, but often failed to increase copy number to the same level in later replicating regions (Figure 6B bottom right). As sync-seq assays a population of cells, we cannot exclude that the observed increased in copy number around origins in *tof1 627* cells, following washout of HU, is due to firing of adjacent dormant origins rather than fork restart. However, we note that copy number increases are usually similar on both sides of early origins following resumption suggesting fork restart rather than dormant origin firing as the cause of increased copy number.

These findings indicate that Tof1 proteins that can mediate DRC activation can promote efficient restart around HU-stalled forks. However, replication forks associated with Tof1 proteins that cannot support replisome coupling or pausing then go on to exhibit elongation defects that often prevent the completion of DNA replication. This leads to toxic lesions if Rad9-dependent repair pathways are absent.

### The fork pausing and replisome coupling functions of Tof1 are linked to its stable interaction with Csm3

**Figure 7.**
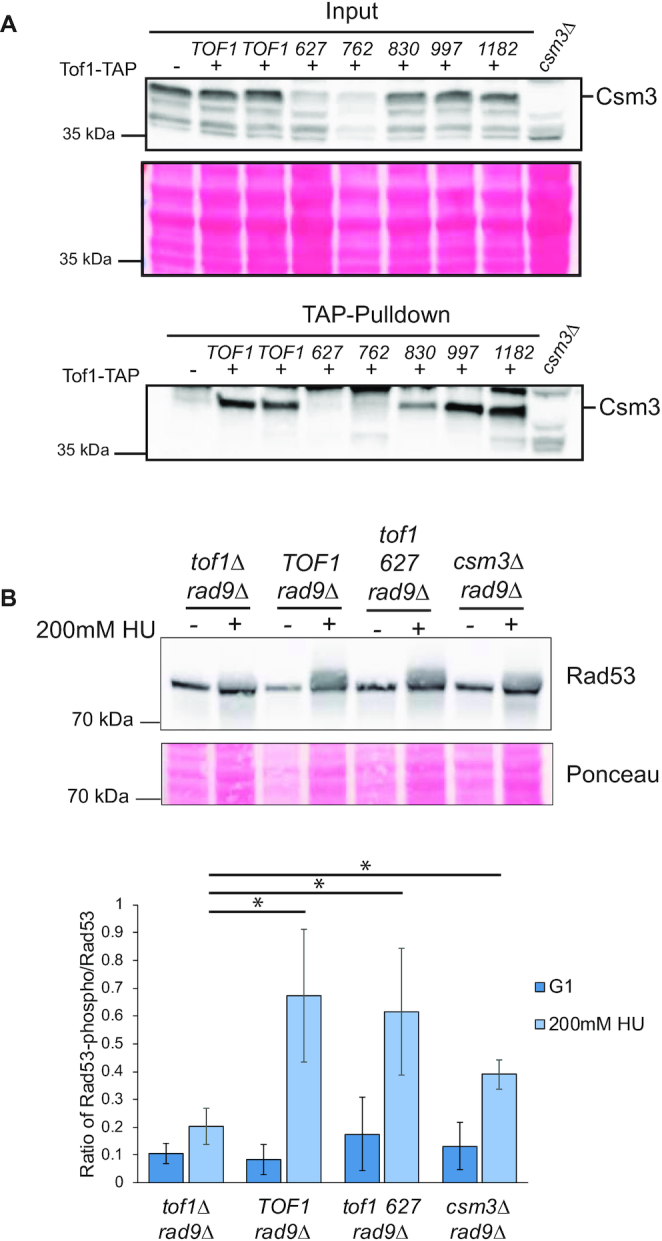
Amino acids 627–830 of Tof1 are required for its stable association with Csm3. (**A**) Pulldown of Csm3 in TAP-tagged Tof1 truncation mutants. Lysates from exponentially growing cells containing the indicated C terminally TAP-tagged *tof1* truncation proteins, were immunoprecipitated and the resulting eluates were western blotted using antibodies to Csm3. Ponceau stain of blotted membrane of the input is shown to illustrate protein content of lanes. Image shown is from one of two equivalent independently conducted experiments. (**B**) The relative mobility of Rad53 in each of the indicated cell types either in exponentially growing cells or following a 2 h treatment with 200 mM HU was assayed by western blot using anti-Rad53 antibodies (upper panel). Ponceau stained membrane of western blot shown in top panel to indicate relative protein levels (middle). The ratio of lower mobility modified Rad53 versus unmodified Rad53 was quantitated from three separate experiments by densitometry of their chemiluminescence. Western blot shown is from one of the three equivalent, independently conducted experiments and the average ratio and standard deviation of the mean are plotted in the histogram (bottom). 200mM refers to concentration of hydroxyurea in the culture. *TOF1 rad9Δ*, *tof1 627 rad9Δ* and *csm3Δ rad9Δ* all generated significantly higher ratios of modified to unmodified Rad53 in response to HU than *tof1Δ rad9Δ* (*P* values: * = <0.05). The apparently lower ratio of Rad53 activation of *csm3Δ rad9Δ* relative to *TOF1 rad9Δ* and *tof1 627 rad9Δ* cells was likely significant (*P* = 0.10 and 0.13 respectively).

In summary, we have found that the function of Tof1 in checkpoint activation only requires the N terminal half of the Tof1 protein. In contrast the roles of Tof1 in supporting fork pausing, suppressing CPT sensitivity, coupling of helicase and polymerase activities and maintaining fork stability following RS all require aa 627–830 of Tof1. The region beyond aa 997 is necessary for Tof1’s role in resolving DNA topological stress ahead of the fork, but is not required for pausing, coupling or resistance to CPT. Recent structural analysis has indicated that the prime interacting interface between Tof1 and Csm3 is within the 627–830 aa of Tof1 (42,43). Therefore, loss of amino acids 627–830 of Tof1 could destabilise the interaction between Tof1 and Csm3. We assessed the stability of the Tof1-Csm3 interaction by immunoprecipitation of TAP-tagged Tof1 truncation proteins and western blotting for Csm3 (Figure 7A). We observed robust interaction between Tof1 and Csm3 in *TOF1*, *tof1 830*, *tof1 997* and *tof1 1182* expressing cells. In contrast we were unable to detect a stable interaction between Tof1 and Csm3 in *tof1 627* and *tof1 762* cells. Previous studies have shown that loss of Tof1/Timeless family proteins leads to a decrease in the detectable level of Csm3/Tipin protein suggesting that the interaction between the two is required for Csm3/Tipin stability in cells (16,44). To assess for loss of Csm3 protein, we western blotted for Csm3 in input extracts of cells expressing WT and truncated Tof1 proteins (Figure 7A). Expression of *TOF1, tof1 830, tof1 997* and *tof1 1182* all supported normal levels of Csm3 protein. Notably expression of *tof1 627* and *tof1 762* led to partially reduced levels of Csm3 in cells (Figure 7A). This is consistent with the *tof1 627* and *tof1 762* truncations compromising the stability of the interaction between Tof1 and Csm3, leading to partial destabilisation of the Csm3 protein.

### Csm3 activity is not necessary for DRC checkpoint signalling

Our phenotypic analysis of the *tof1 627* and *tof1 762* truncations predicts that the Tof1-Csm3 interaction is not essential for DRC activation, since both mutations are proficient for activation but do not stably interact with Csm3. To test this prediction, we compared the level of DRC activation in *tof1Δ rad9Δ, tof1 627 rad9Δ and csm3Δ rad9Δ* cells by visualisation of Rad53 phosphorylation in these cells following acute HU treatment. We observed that Csm3 is not required for DRC activation in *rad9Δ* cells, as c*sm3Δ rad9Δ* cells were significantly more proficient in mediating Rad53 phosphorylation than *tof1Δ rad9Δ* cells (Figure 7B). We did note a likely reduction in activation compared to cells expressing *TOF1* and *tof1 627* (*P* = 0.10 and 0.13 respectively). We conclude that Csm3 is not essential for DRC activation but that its presence likely stabilises the DRC proficient conformation of the replisome.

## DISCUSSION

Defining how the Timeless protein functions to maintain genome stability, both generally and following RS has been complicated by its multiple functions. Here we have shown that distinct regions of Tof1 are required for its roles during DNA replication.

We find that the far C terminal region (>997aa) of Tof1 is required to suppress excessive fork rotation in cells. This region is also sufficient to mediate a two-hybrid interaction with the type 1B topoisomerase Top1 (23) and is required for the *in vivo* interaction of Tof1 and Top1 (24) (this study). During DNA replication Top1 can only act ahead of the replication fork to facilitate unwinding of the parental DNA duplex (3,22). Therefore, the recruitment of Top1 to the replisome via the C terminal region of Tof1 will bias topoisomerase action to the region ahead of the fork. This accounts for the requirement of this region to suppress the alternate pathway of duplex DNA unwinding, specifically fork rotation and the action of Type II topoisomerase behind the fork. During preparation of this study others have also shown that a similar C terminal region of Tof1 is required to recruit Top1 to active replication forks, consistent with this model (24). Interestingly, the SV40 T antigen helicase also directly recruits Top1 to the SV40 replisome to facilitate DNA unwinding (45) suggesting that direct replisome recruitment of Top1 could be a general feature of eukaryotic replisomes. It remains to be seen if topoisomerase recruitment to the eukaryotic replisome is normally achieved via Timeless family proteins or if this function could be swapped between different replisome components in different organisms.

Distinct from the far C terminus, the region of Tof1 between amino acids 627 and 830 is required for fork pausing at the RFB protein barrier and also to maintain coupling of helicase and polymerase activities during HU induced RS. Expression of *tof1 627* and *tof1 762* truncations ablates the interaction between Tof1 and Csm3, consistent with recent structural analyses of this heterodimer (42,43). Loss of this region appears to be linked to a general reduction of Csm3 protein levels in cells, suggesting that the interaction is required to stabilise Csm3 in cells. We speculate that the Tof1–Csm3 heterodimer plays a central role in replisome structure that facilitates coordination of CMG and replicative polymerases consistent with observations across several systems (14,15,17). Current data argues that functional consequences of the Tof1–Csm3 interaction extend beyond the mapped 627–830 aa region of Tof1. For example, we have previously shown that *csm3Δ* cells do not suppress excessive fork rotation, despite full length Tof1 still being expressed (22). Loss of Csm3 also reduces the association of Mrc1 with the replication fork (16). This argues that the Tof1–Csm3 interaction is required to appropriately configure the Mrc1–Tof1 and Tof1–Top1 interactions within the replisome. The Csm3 interacting region of Tof1 is also required to suppress the sensitivity of *tof1Δ* cells to the Top1cc complexes generated by CPT. Since the C terminal region required for the Tof1–Top1 interaction is dispensable for CPT resistance, it appears that the coordination of Top1 action with the replisome by Tof1 is dispensable for normal cellular responses to CPT. Rather our data argue that CPT sensitivity is closely linked with stable fork pausing and helicase-polymerase coupling. We speculate that Tof1 activity allows the fork to pause when encountering a Top1cc complex on the leading strand, preventing polymerase runoff and the generation of a potentially lethal single ended DNA DSB (31).

While our data show that the C terminal region beyond aa 830 is not required for fork pausing, coupling and resistance to CPT, we have also found that disruption of the polypeptide structure beyond aa 830 can affect these functions. Further work is required to investigate if this effect is pathological or indicative of a regulatory function for the C terminus. Whatever the exact nature of this effect, it does provide an explanation of the difference between the phenotypes we describe here and those described for C terminally tagged Tof1 proteins truncated at aa 981 (24).

Finally, we find that the N-terminal half (627 aa) of Tof1 is sufficient for intra-S-checkpoint signalling in response to HU as assayed by the Rad53 phospho-shift assay. Both Mrc1 and Tof1 are required for efficient checkpoint signalling in HU (9,10). In many eukaryotic systems, depletion of Tipin also ablates DRC signalling (17,44). Here, we show that neither the stable interaction of Tof1-Csm3, nor even the presence of any cellular Csm3, is necessary for DRC signalling. We speculate that this difference is due to the different levels of Tof1/Timeless protein expressed in cells following loss of Csm3/Tipin in different organisms. Tof1 protein is apparently stable in *S. cerevisiae* in the absence of Csm3 (16) whereas in other systems loss of Tipin results in co-depletion of Timeless protein (17,44). During DRC activation the N terminal half of Tof1 could either have a direct role in promoting Rad53 activation or an indirect role in DRC activation by appropriately orientating Mrc1 in the replisome. Since Mrc1 alone appears capable of facilitating the sensor/effector kinase interaction (46), and Tof1 is required to promote association of Mrc1 with the replisome (12,16), we favour the later scenario and we speculate that the N terminal half of Tof1 is required to functionally orientate Mrc1 at the replication fork to promote DRC activation. Consistent with this model, cryo-EM modelling of the replisome indicates that the interface between Tof1 and Mrc1 occurs across the N terminal half of Tof1 (42). Since complete loss of Csm3 likely leads to some reduction in the phospho-shift of Rad53 protein, our results suggest that Csm3 has a role in stabilising the N terminal Tof1 dependent, DRC signalling competent, replisome conformation of Mrc1. This would be consistent with loss of Csm3 causing reduced chromatin association of Mrc1 in ChIP assays (16).

DRC activity following fork stalling ensures that the replisome and fork structure is maintained in a form capable of efficiently restarting. Tof1’s role as a mediator protein has provided a straightforward explanation as to why its absence results in problems in fork restart (11). However, the general replisome instability of *tof1Δ* cells also suggests that the replisome structure formed in the absence of Tof1 may not be proficient in replication following restart after RS. The phenotype of the separation of function mutation *tof1 627*, which is proficient for checkpoint signalling but not proficient for replisome coupling or fork pausing, indicates that Tof1 has important roles in completing DNA replication following RS beyond purely mediating DRC activation. Although *TOF1* and *tof1 627* cells appear to have equivalent levels of Rad53 mobility shift, we cannot exclude the possibility that the cellular activity of the DRC may be different in the two conditions. Nevertheless, our data argues that Tof1 functions separate from DRC activation are required to stabilise replication forks following resumption of DNA replication after RS. The reason for fork failure following resumption of DNA replication is unclear but we speculate that *tof1* mutants deficient in coupling helicase and polymerase activity in HU are also defective in efficient recoupling during recovery from HU treatment, leading to frequent fork failure during elongation. Notably, the sensitivity of *tof1* mutants to HU is highly dependent on the activity of Rad9, consistent with Rad9 dependent repair pathways being required to resolve *tof1* linked DNA lesions. Although we show that Tof1 has roles in recovering from RS beyond mediation of the DRC, we cannot rule out a role for Tof1 downstream of DRC activation. The phosphorylation state of Tof1 is partially dependent on Mec1/Tel1 activity (47) and it is known that Tof1 phosphorylation can influence fork pausing (48). Therefore, checkpoint dependent phosphorylation of the C terminal half of Tof1 could be important for recovery of the fork after RS.

In summary, our data argues that the N-terminal half of Tof1 is linked to DRC functions, potentially through association with Mrc1, that the far C terminus regulates Top1 association with the replisome and that the region of Tof1 between aa 627 and aa 830 is required for stable binding to Csm3 (Supplementary Figure S5). These data indicate that Tof1 has roles both in co-ordinating general replisome architecture through separate Mrc1 and Csm3 interactions, as well as specifically recruiting other activities such as Top1 to the replisome.

## DATA AVAILABILITY

All raw and processed data are available at the Gene Expression Omnibus (GEO) under the accession number: GSE144321 (https://www.ncbi.nlm.nih.gov/geo/query/acc.cgi?acc=GSE144321)

The data on UCSC can be reached on this link:

https://genome.ucsc.edu/s/a.keszhelyi/Tof1_Westhorpe.

## Supplementary Material

gkaa963_Supplemental_File
