# Supplementary material for: Separable functions of Tof1/Timeless in intra-S-checkpoint signalling, replisome stability and DNA topological stress
Source: Nucleic Acids Res. 2020 Nov 9;48(21):12169–87. doi: 10.1093/nar/gkaa963 (PMC7708041; doi:10.1093/nar/gkaa963)
Supplement: gkaa963_Supplemental_File [file gkaa963_supplemental_file.pdf]

Supplementary Figure 1

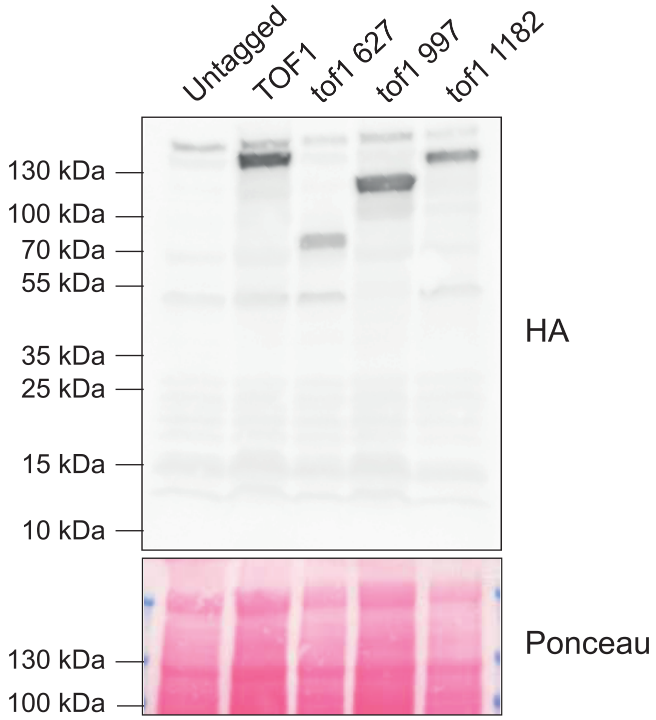

Supplementary Figure 1: Expression of C-terminal Tof1 truncation mutations using an N terminal HA epitope tag

TOF1, tof1 627, tof1 997 and *tof1 1182* were N-terminally tagged with HA epitope and lysates were checked for expression using antibodies against the HA epitope. Ponceau stain of blotted membrane is shown to illustrate protein content of lanes. Image shown is from one of two equivalent, independently conducted, experiments.

Supplementary Figure 2

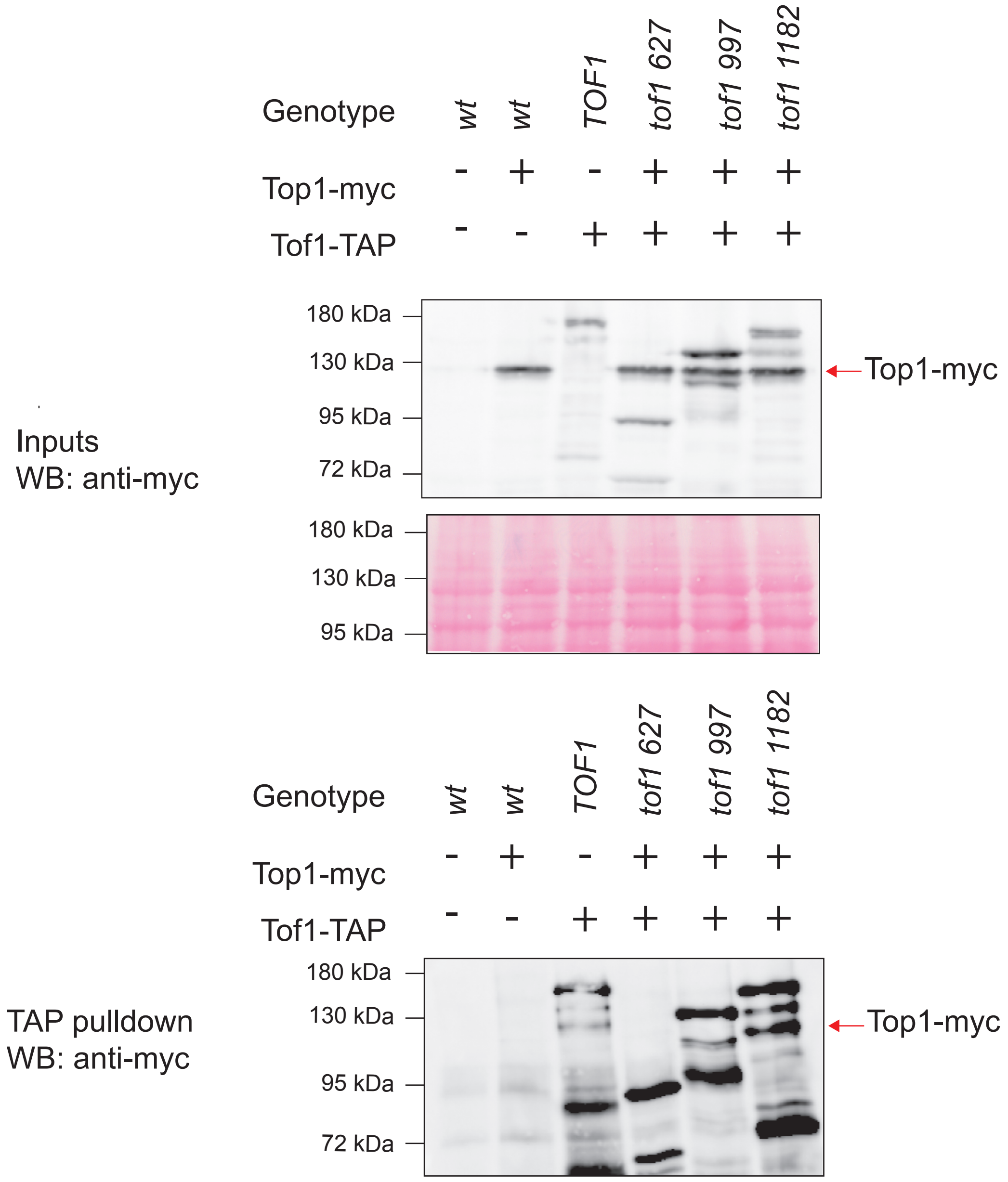

Supplementary Figure 2: Amino acids 997-1182 of Tof1 are required for its interaction with Top1

Extracts from cells expressing *tof1 627, tof1 997* and *tof1 1182* tagged on the C terminus with with TAP epitope in cells and Top1 C terminally tagged with the myc epitope were used immunoprecipitated with using IgG resin to isolate TAP tagged proteins. The resulting eluates were western blotted using antibodies to the myc epitope. 1% input of extract is blotted with antibodies to myc in the top panel while pulled down proteins are probed in the bottom panel. The ponceau stained membrane of the extract blot is shown to compare lane loading. Images shown are from one of two equivalent experiments.

Note, that the different sized TAP tagged Tof1 truncated protein are also detected by the IgG used to detect the myc epitope in both input and eluate samples. Cells containing Top1-myc alone were probed in the input blot to demonstrate the exact mobility of Top1-myc relative to the various Tof1-TAP proteins detected by the anti myc antibody.

Supplementary Figure 3

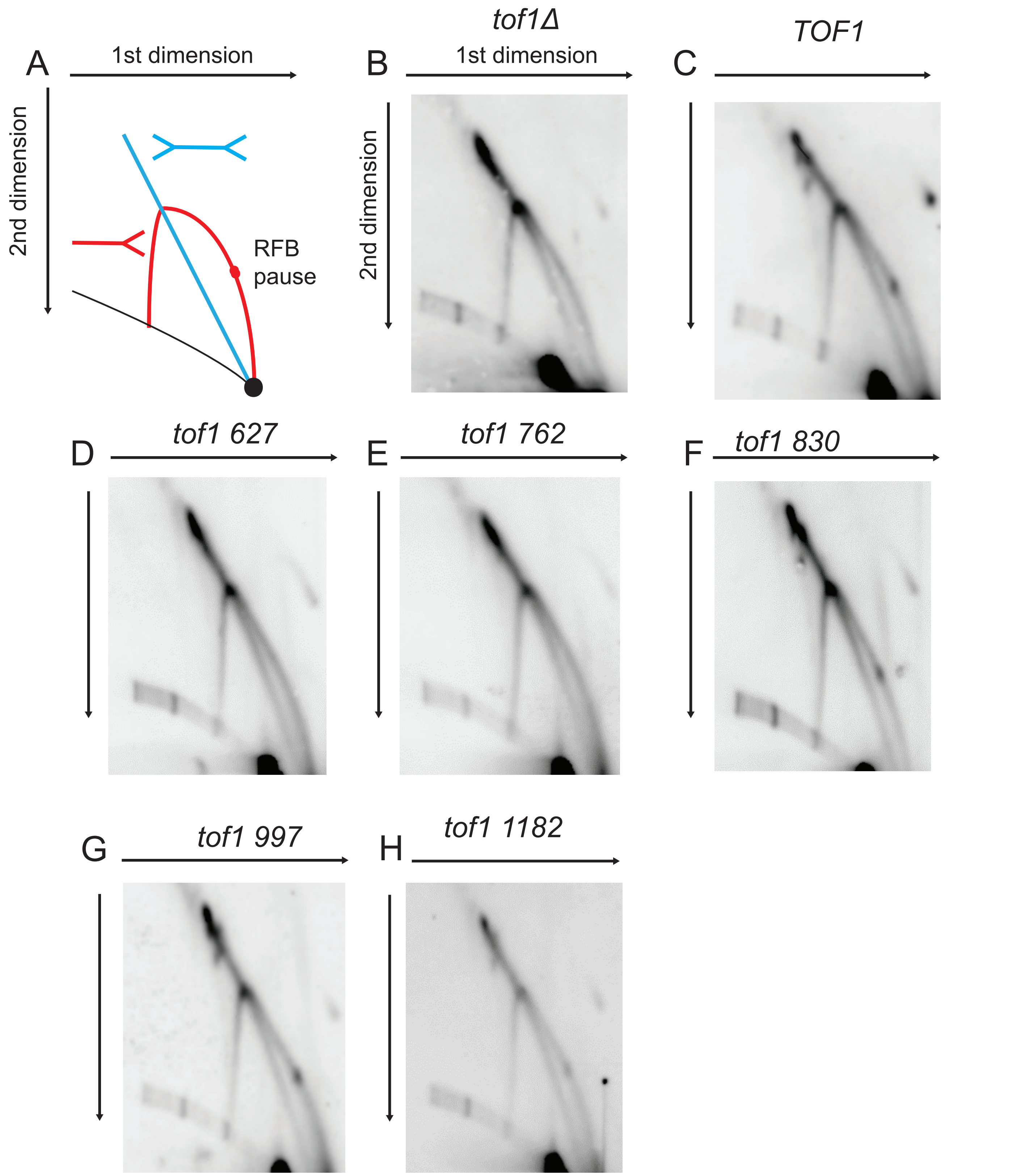

Supplementary Figure 3: Two dimensional gel analysis of SnaB1 digested pRS426-RFB in tof1 C terminal truncation mutants.

A) Schematic of the replication fork Y arc predicted to be generated by pRS426-RFB digestion with SnaBI in wildtype cells and resolved by 2D gel electrophoresis. Arrow indicates the accumulation of replication intermediates generated by pausing at the RFB on pRS426.

B-H) Representative image of one of two replicates of 2D gel analysis of SnaBI digested pRS426-RFB extracted from exponentially growing cells containing

B) *tof1Δ* C) *TOF1* D) *tof1 627* E) *tof1 762*

F) *tof1 830* G) *tof1 997* H) *tof1 1182*

Supplementary Figure 4

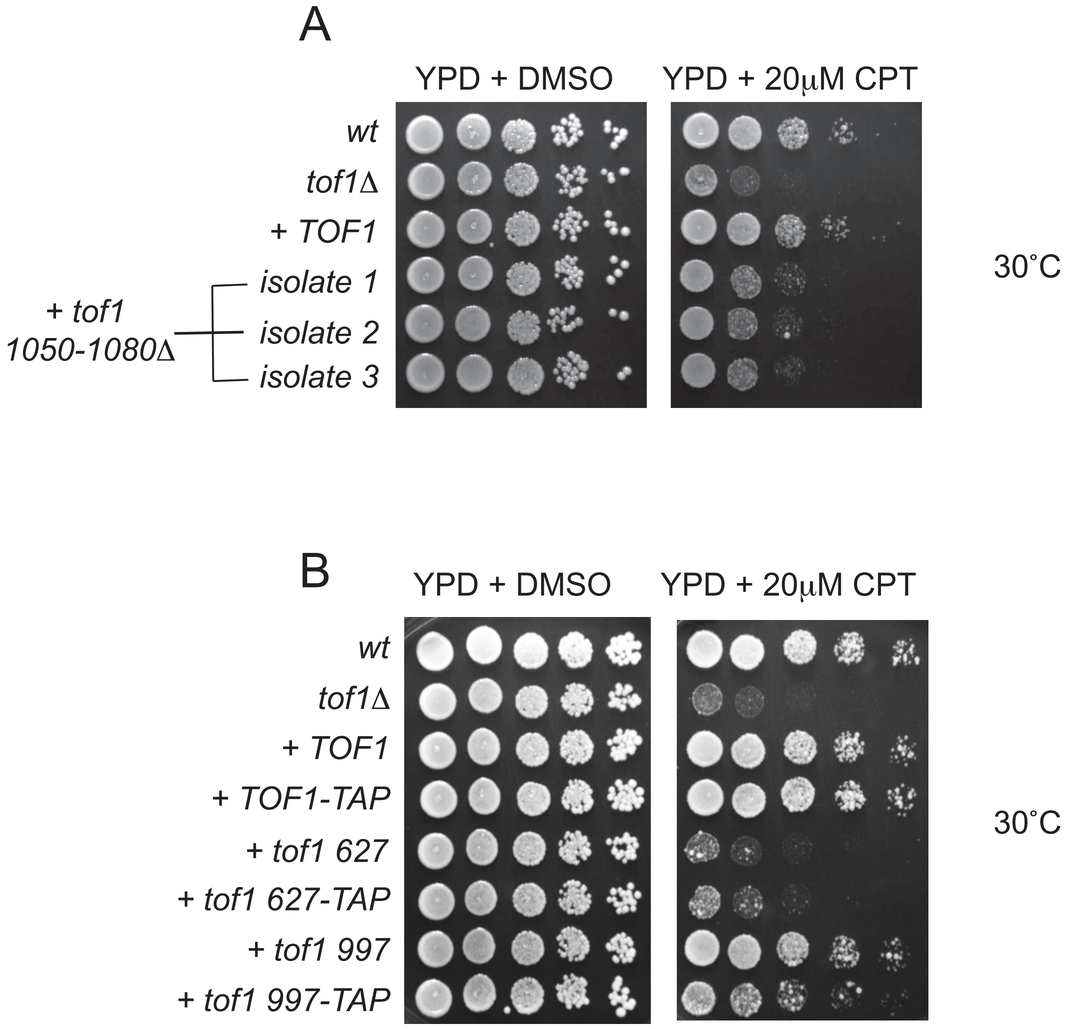

Supplementary Figure 4: Deletion within the C terminal region or addition of C terminal epitope to the C terminus of Tof1 can cause CPT sensitivity.

A) Viability spot assay of wildtype (wt), *tof1Δ, TOF1* and three separate isolates of *tof1 1050-1080*Δ. Cells were spotted in ten-fold sequential dilutions on either YPD + DMSO or YPD plus 20 μg/ml camptothecin plates and incubated for 48 hours at 30°C.

B) Viability spot assay of wildtype (*wt*), *tof1Δ*, and*TOF1*, *tof1 627, tof1 997* cells both without and with addition of a C terminal TAP tag. Cells were spotted in five-fold sequential dilutions on either YPD plus DMSO or YPD plus 20 μg/ml camptothecin plates for 48 hours at 30°C.

Supplementary Figure 5

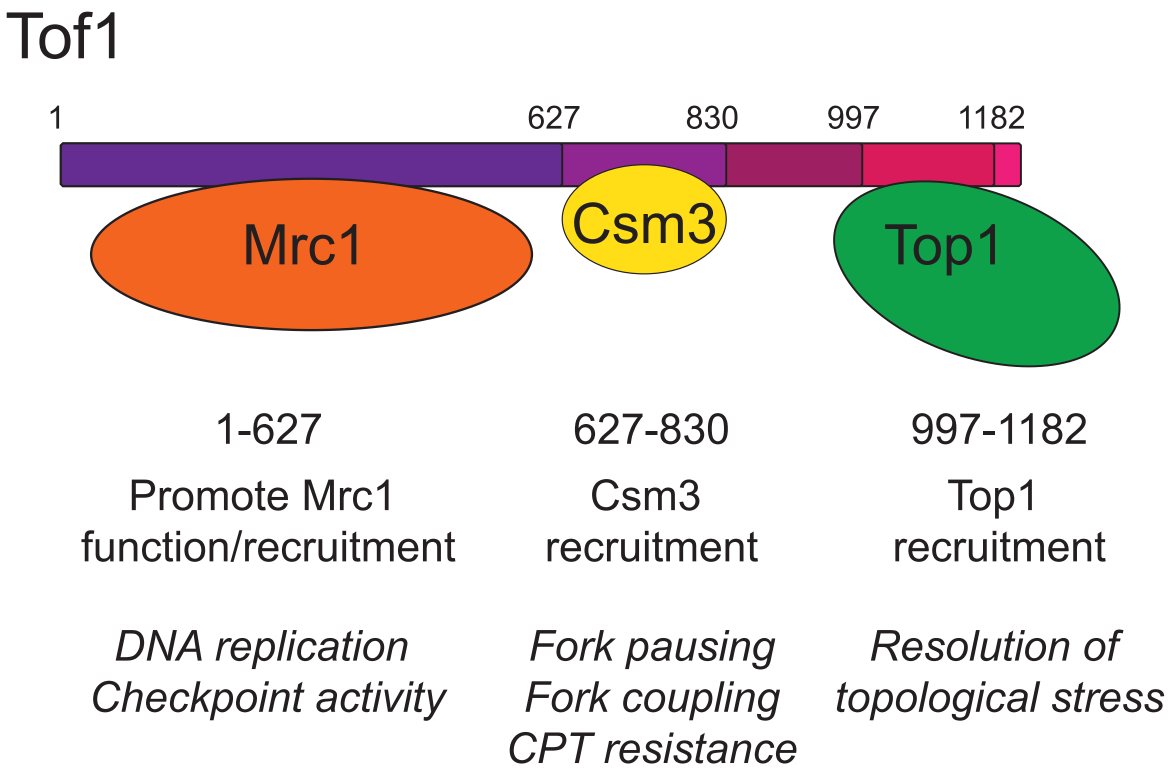

Supplementary Figure 5: Summary of the Tof1 functional domains required for distinct replisome processes during DNA replication

The N terminal half of Tof1, specifically residues 1-627, promote DRC activities potentially by recruitment and/or co-ordination of Mrc1 function in checkpoint mediation.
Residues 627-830, are required for Csm3 binding and promote replisome pausing and stabilisation functions, most likely by properly co-ordinating Csm3 ahead of the replisome.
The far C terminal domain between residues 997-1182 of the protein promotes resolution of supercoiling ahead of the progressing CMG through its interaction with Top1.
